# Respiratory Inductance Plethysmography to Quantify Changes in Ventilation in Obstructive Sleep Apnea

**DOI:** 10.1109/TBME.2025.3618403

**Published:** 2026-05

**Authors:** Eysteinn Finnsson, Eydís Arnardóttir, Kristófer Montazeri, Brendan T. Keenan, Richard J. Schwab, Thorarinn Gislason, Allan I. Pack, Andrew Wellman, Anna S. Islind, Jón S. Ágústsson, Scott A. Sands

**Affiliations:** Nox Research, Nox Medical ehf, Reykjavik, Iceland and Reykjavik University, Department of Computer Science, Reykjavik, Iceland.; Nox Research, Nox Medical ehf, Reykjavik Iceland.; Nox Research, Nox Medical ehf, Reykjavik Iceland.; Division of Sleep Medicine, Department of Medicine, Perelman School of Medicine, University of Pennsylvania, Philadelphia, PA, USA.; Division of Sleep Medicine, Department of Medicine, Perelman School of Medicine, University of Pennsylvania, Philadelphia, PA, USA.; Department of Sleep, Landspitali - The National University Hospital of Iceland, Reykjavik, Iceland and Faculty of Medicine, University of Iceland, Reykjavik, Iceland; Division of Sleep Medicine, Department of Medicine, Perelman School of Medicine, University of Pennsylvania, Philadelphia, PA, USA.; Division of Sleep and Circadian Disorders, Department of Medicine, Brigham and Women’s Hospital and Harvard Medical School, Boston, MA, USA.; Reykjavík University, Department of Computer Science, Reykjavík, Iceland.; Nox Research, Nox Medical ehf, Reykjavik, Iceland; Division of Sleep and Circadian Disorders, Department of Medicine, Brigham and Women’s Hospital and Harvard Medical School, Boston, MA, USA.

**Keywords:** event depth, minute ventilation, obstructive sleep apnea, oral breathing, respiratory inductance plethysmography, ventilatory burden

## Abstract

**Background and objective:**

The study aims to determine whether respiratory inductance plethysmography (RIP) signals can be used to quantify changes in ventilation and provide advanced obstructive sleep apnea (OSA) severity metrics. This approach seeks to address limitations in current airflow-based OSA measures, particularly those relying on nasal pressure, which may be compromised by oral breathing.

**Methods:**

Adult patients with OSA (N=89, 68Male:21Female) completed in-laboratory polysomnography (PSG) allowing for RIP-based ventilation estimates to be compared against a gold standard oronasal-pneumotach (normalized ventilation %_eupnea_). Concordance was assessed on three levels: 1) individual breath ventilation, 2) individual respiratory event depth (percentage reduction in ventilation from local average), and 3) patient-specific OSA severity in terms of average event depth and ventilatory burden (average event depth × average event duration × event rate). To address overestimation of RIP ventilation during obstruction, we developed and applied a calibration and linearization method (“RIP correction”). Concordance analysis evaluated median bias for both small (<70%_eupnea_) and large breaths (>130%_eupnea_), along with bias and intraclass correlation coefficient (ICC) calculation for events and patient-specific measures.

**Results:**

For individual breaths (N=495,631), RIP correction reduced overestimation bias for small breaths from 12 to 2 %_eupnea_. For individual events (N=34,497), RIP correction reduced mean bias for event depth estimates from 9 to 1 %_eupnea_. For patient-specific analysis underestimation of average event depth was attenuated from 9 to 4 %_eupnea_ and for ventilatory burden, from 275 to 116 %_eupnea_ min/hr. Additionally, RIP correction improved ICC for event depth and patient-level traits.

**Conclusion:**

RIP signals, with appropriate processing, enable quantification of advanced ventilation-based OSA metrics, addressing concerns that airflow-based measures may be affected by breathing route.

## I. Introduction

Obstructive sleep apnea (OSA) is a highly prevalent disorder characterized by recurrent loss of ventilation during sleep; the resultant arterial hypoxemia and sympathetic activation have major consequences for daytime health, wellbeing, and quality of life [1], [2], [3], [4]. OSA is diagnosed using signals collected by polysomnography (PSG). These include electroencephalogram to monitor brain activity, electrooculogram for eye movements, electromyogram for muscle activity, and respiratory inductance plethysmography (RIP) for abdomen and chest movements. PSG also includes monitoring oxygen saturation levels using pulse oximetry and airflow using a nasal cannula coupled with a pressure transducer. The diagnosis of OSA is based on the rate of discrete breathing events during the night. A complete cessation of airflow for at least 10 seconds is defined as apnea and hypopnea is defined as a reduction of airflow by at least 30% for a minimum of 10 seconds, accompanied by either a 3% or more drop in oxygen saturation or a cortical arousal [5]. Mouth breathing is common among patients with OSA, who typically spend a larger portion of the night breathing orally compared to those without OSA [6], [7]. The reliance on the nasal cannula and a lack of oral airflow measurement in standard PSG may result in systematic errors in those patients. Oronasal thermistors are recommended by the American Academy of Sleep Medicine (AASM) for detecting apneas [5], particularly in the presence of oral breathing, but their output is suboptimal for quantitative analysis because it is not proportional to airflow [8]. Since hypopneas are scored from nasal pressure signals, partial oral breathing may go undetected, and thermistors are not recommended for this purpose. Thus, mouth breathing can confound both apnea and hypopnea scoring, potentially altering OSA assessment. Despite this relevance, the literature on its clinical impact remains limited, in part because concurrent measurement of oral and nasal airflow is technically challenging and has therefore been confined to small physiological studies [9]. Meanwhile, a precise measure of airflow during sleep can be employed for a variety of applications. For example, this enables advanced OSA analyses, such calculating average event depth, which assesses upper airway collapsibility by measuring the reduction in ventilation relative to a local baseline [10], and ventilatory burden, a composite severity metric that integrates event depth, duration and frequency into a single measure [11]. Both measures use a minute ventilation signal, obtained by integrating flow per breath to volume, dividing by breath duration, and normalizing to a local eupneic baseline. By this definition, apnea corresponds to 0% eupnea and normal unobstructed breathing to 100% eupnea.

RIP is a promising alternative signal for the clinical assessment of airflow, as it provides a surrogate measure of breathing through abdominal and thoracic movement. Similar to the nasal cannula, RIP is part of a standard PSG and is recommended by the AASM as a secondary airflow sensor [5]. RIP estimates changes in the circumference of the abdomen and thorax, which reflect the changes in tidal volume. High quality RIP has been shown to be capable of providing a reliable measure of airflow [12]. However, to derive an airflow signal from breathing movements, the RIP signals need to be calibrated [13]. Calibration allows the measurements from each belt to be summed to get a signal that is proportional to tidal volume [14]. The need for RIP calibration is most evident during periods of obstructive apnea, where the airway is completely obstructed, and the abdomen and thorax exhibit an out-of-phase movement (i.e., paradoxical breathing). Failing to properly calibrate the RIP signals during these periods results in a residual movement signal and an overestimation of airflow.

In this study, we introduce a method for using RIP to quantify relative changes in airflow. We develop algorithms to calibrate and linearize the RIP signals, providing a more accurate estimate of tidal volume and flow. Using these signals, we derive both minute ventilation and associated endotypic traits of OSA, specifically, average event depth [10] and ventilatory burden [11]. We then compare them with the same variables derived from an oronasal pneumotachograph. We hypothesize that our method will improve the derivation of these variables compared to existing calibration approaches.

### II. Methods

### A. Design

The current study was a secondary analysis of a larger, multicenter, observational cohort study named: *Mechanisms of Extreme Phenotypes in Obstructive Sleep Apnea* (*EXPO)* (NIH project number 5P01HL094307–10). Adult male and female participants were recruited from the Hospital of the University of Pennsylvania, USA and Landspítali University Hospital, Iceland (IRB protocol number 823038 and VSN-17–049). Participants provided written-informed consent before participation. The study included participants aged 18 to 80 years with either an apnea-hypopnea index (AHI) ≥ 15 or ≤ 5 events/hour, and a body mass index (BMI) ≥ 35 or ≤ 25 kg/m^2^, who had a stable medical history and adhered to pre-study restrictions regarding alcohol and sleep apnea treatments. The study excluded patients with sleep disorders other than OSA, recent surgeries or medical instability, certain medication uses, metal implants, and pregnancy; see Supplementary Material for details.

### B. Participants

From the data available at the time of the current study, 99 scored polysomnographic recordings were obtained from patients with diagnosed OSA (i.e., AHI ≥ 5 events/h) and adequate total sleep time (TST ≥ 4 hours). Of these, eight studies were excluded due to poor, or missing oronasal flow and two were excluded due to connection fault of the abdominal RIP belt, leaving data from 89 patients for analysis.

### C. Polysomnography

Studies were recorded with the Nox A1 (Nox Medical, Iceland), which included abdominal and thoracic RIP (unfiltered, true inductance technology for the full circumference). In place of the nasal cannula, usually included in clinical PSG, quantitative oronasal ventilatory flow was measured by pneumotach via a sealed oronasal mask (Hans Rudolph, Shawnee, Kansas, USA). RIP belts were positioned according to standard clinical in-lab practice. The thoracic belt was placed below the armpits and above the breasts—at or above nipple level—and the abdominal belt at the level of the umbilicus. As the belts were not taped or otherwise secured, they were susceptible to displacement during sleep. The sleep studies were manually scored by a trained sleep technician in accordance with AASM scoring guidelines [5].

### D. Breath detection

Breath detection was performed with two separate algorithms depending on the underlying signal. For the RIP signals, breaths were detected by identifying concurrent movement in the abdominal and thoracic signals, accounting for both normal (in-phase) and paradoxical (out-of-phase) breathing. During central apneas, breaths were imputed from the pre-event respiratory rate. For the oronasal flow signal, breaths were detected by removing the baseline with a moving-window median filter (1-minute window), integrating the signal to obtain volume, and identifying troughs in this volume signal as breath onsets. During apneas, breaths were imputed from the pre-event respiratory rate.

### E. RIP Ventilation Analysis

**Fig. 1.**
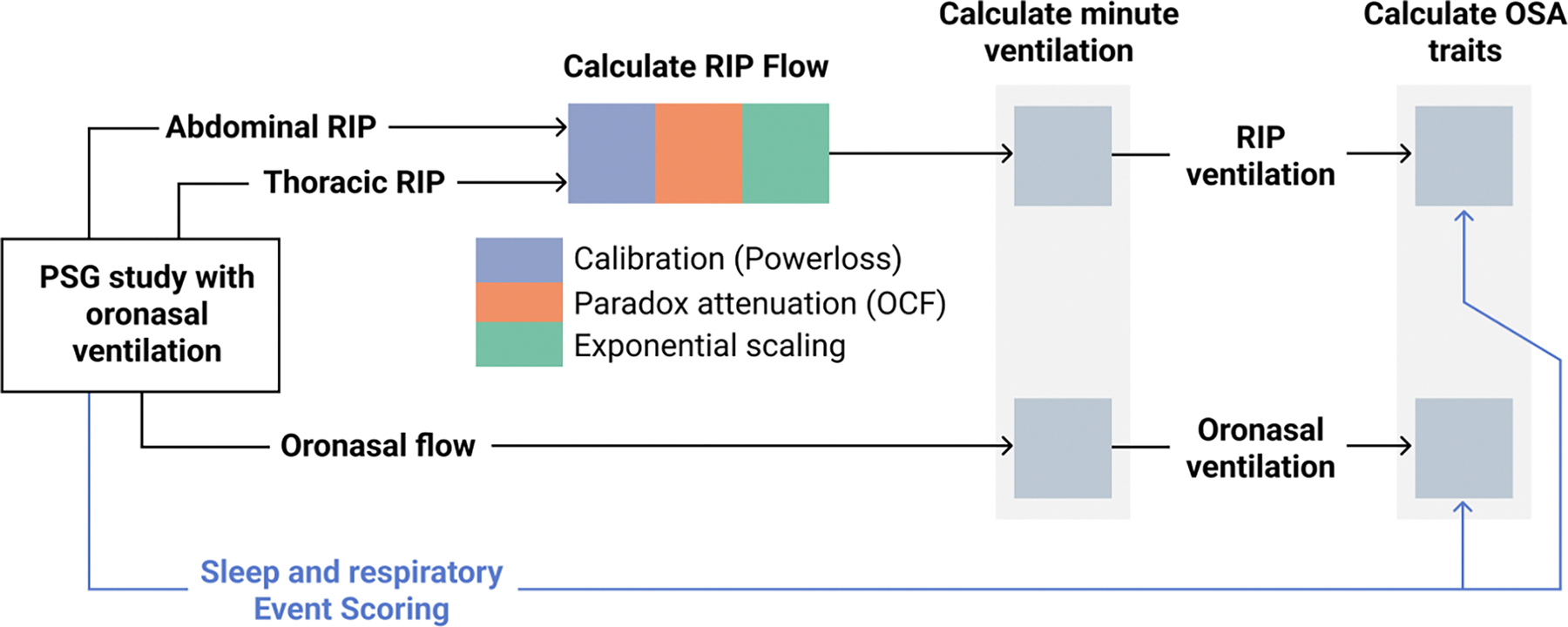
Flow diagram showing how ventilation and traits were calculated using RIP and oronasal pneumotach flow. For each sleep study the RIP signals and oronasal pneumotach signal are processed independently. From these signals we derive two sets of data: minute ventilation and obstructive sleep apnea (OSA) traits. The traits we focus on in this study are average event depth and overall ventilatory burden.

We developed an approach for estimating ventilation from RIP on a breath-by-breath basis during sleep. Fig. 1 outlines the key processing steps, which are further detailed below. Signal processing analyses were performed using Python 3.11, with NumPy 1.23.5, Matplotlib 3.8.0 and SciPy 1.11.3 [15], [16], [17].

### F. Calculating RIP Flow: RIP correction algorithm

To optimize the estimation of ventilation using the RIP signals, three processing steps were applied in sequence, as shown in Fig. 1. These processing steps are referred to collectively as “RIP correction”, and are described as follows:

#### 1. Linear calibration of the RIP signals using the ‘powerloss’ [18] method.

Linear RIP calibration aims to find a factor, k, that scales the abdominal (RIP_ab_) and thoracic (RIP_th_) breathing movement signals such that their sum becomes proportional to tidal volume according to: Tidal Volume = (1-k) RIP_th_ + k RIP_ab_. A flow signal (RIP_flow_) can then be calculated by taking the time derivative of the tidal volume signal. The powerloss method attempts to find the optimal k by minimizing the residual variance in the calibrated RIP flow signal caused by paradoxical movements in the RIP belts; see Equation 1 where dRIP is the time derivative of the respective RIP signal, RMS is the Root Mean Square function and k takes a value between 0 and 1. In the limit where breathing is unobstructed, and no paradoxical movements are present, the powerloss method is analogous to the Qualitative Diagnostic Calibration (QDC) method, which estimates calibration factors during natural breathing by normalizing the relative variance of thoracic and abdominal tidal excursions [13], and in the limit where the airway is completely obstructed the powerloss method becomes equivalent to Isovolume Calibration (ISOCAL), which determines calibration factors by exploiting isovolume maneuvers where thoracic and abdominal excursions are equal and opposite [14].

(1) $$\underset{k}{min}\frac{RMS\left((1\;-\;k)\;\times \;dRIP_{th}\;+\;k\;\times \;dRIP_{ab}\right)}{RMS\left((1\;-\;k)\;\times \;dRIP_{th}\right)\;+\;RMS\left(k\;\times \;dRIP_{ab}\right)}$$

To allow for recalibration when a patient moves, k was updated using an automated adaptive strategy. This approach divided the night into multiple constant calibration periods, which were separated by movement events. These movements were identified by detecting a change in the baseline RIP values that exceeded 15 times the median tidal volume. The detection process involved comparing the measured RIP signal to a 20-breath long reference pattern. These values were chosen pragmatically to balance stability and responsiveness and were validated visually. A single value for k was maintained constant within each period.

#### 2. Detection and suppression of residual breathing movements in the RIP tidal volume signal during obstructed breathing.

**Fig. 2.**
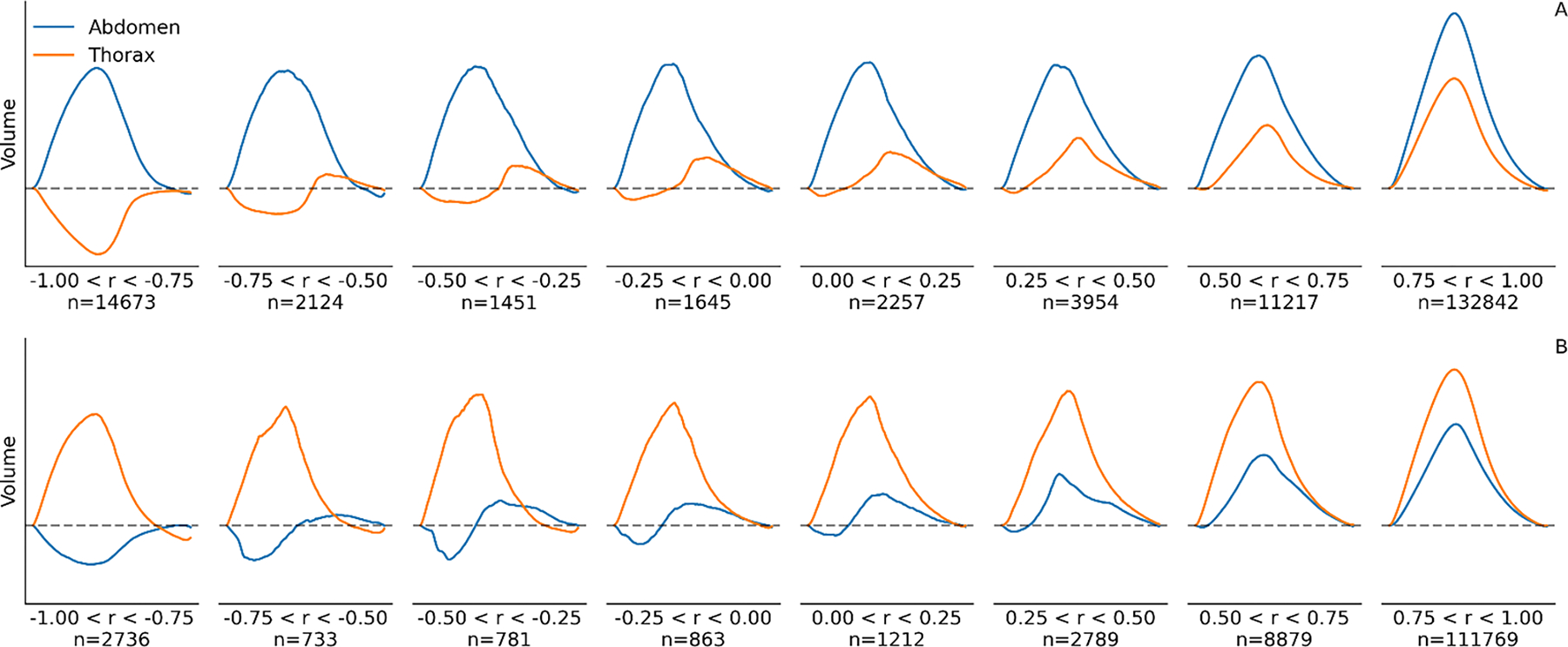
RIP movement patterns across correlation levels. This set of plots represents averaged respiration patterns categorized by ranges of synchrony between abdominal and thoracic movements, as measured by correlation coefficient (r). Plot A shows breaths where the abdomen (blue line) is the primary driver of ventilation, while in B the thorax (orange line) is dominant. The correlation values span from −1.00 (indicative of completely paradoxical movement) to 1.00 (representing perfectly synchronous movement). The volume waveforms for each belt are plotted for different r value ranges, with each panel showing how many breaths (n) were aggregated to create the plot for the corresponding range. This visualization highlights the transition from paradoxical to synchronous respiratory motion as the correlation coefficient increases

Despite optimal linear calibration, abdominal and thoracic excursions describe just two compartments of a more complex respiratory system. We recognize that, during apneas, paradoxical breathing movements are generally not perfectly canceled out, leading to residual breathing movements in the RIP tidal volume signal. This results in a systematic overestimation of RIP ventilation during obstructive apneas. To quantify and correct this expected cause of error, the following approach was applied: The level of paradoxical movement in each breath was gauged by calculating the Pearson correlation coefficient (r) between the abdominal and thoracic RIP signals, as this metric directly captures the degree of linear synchrony between the two signals. When no paradoxical movement is present, r should take a value of 1, while the value should be −1 during complete out of phase movement of the two RIP signals; see Fig. 2. To quantify how overestimation in ventilation relates to r, the ratio of oronasal ventilation to RIP ventilation was calculated for all breaths (all patients pooled) and binned into deciles based on r values. A sigmoid was fitted to the binned data to derive an overestimation correction factor (OCF); See Figure S1 in the Supplementary Material. The resulting Equation 2 where $x_{0}=0.034$, k=31.0 and $y_{\text{min}}=0.22$ is used as a function to correct the systematic bias by suppressing the RIP flow signal amplitude during paradoxical movements, see Equation 3 and Figure S2 in the Supplementary Material.

(2) $$OCF(r)=\left(1-y_{min}\right){\left(\frac{1}{1\;+\;e^{-k\left((r+1)/2-x_{0}\right)}}\right)}^{2}+y_{min}$$

(3) $$RIP_{\mathrm{flow}}^{\prime }=RIP_{\mathrm{flow}}\times OCF\left(corr\left(RIP_{ab},RIP_{th}\right)\right.$$

#### 3. Correction for overestimation of tidal volume in small breaths and underestimation of tidal volume during large breaths.

In the current data, we observed a nonlinear relationship between oronasal ventilation and RIP ventilation. Namely, RIP ventilation tended to underestimate larger breaths while overestimating the smaller ones. To correct for this, a power-law correction factor was optimized using the ventilation values from a subset of the study data and used to scale the RIP ventilation in a similar approach employed by Sands et al. [19] for linearizing the nasal cannula; RIP_flow_ = RIP_flow_^1.17^.

**Fig. 3.**
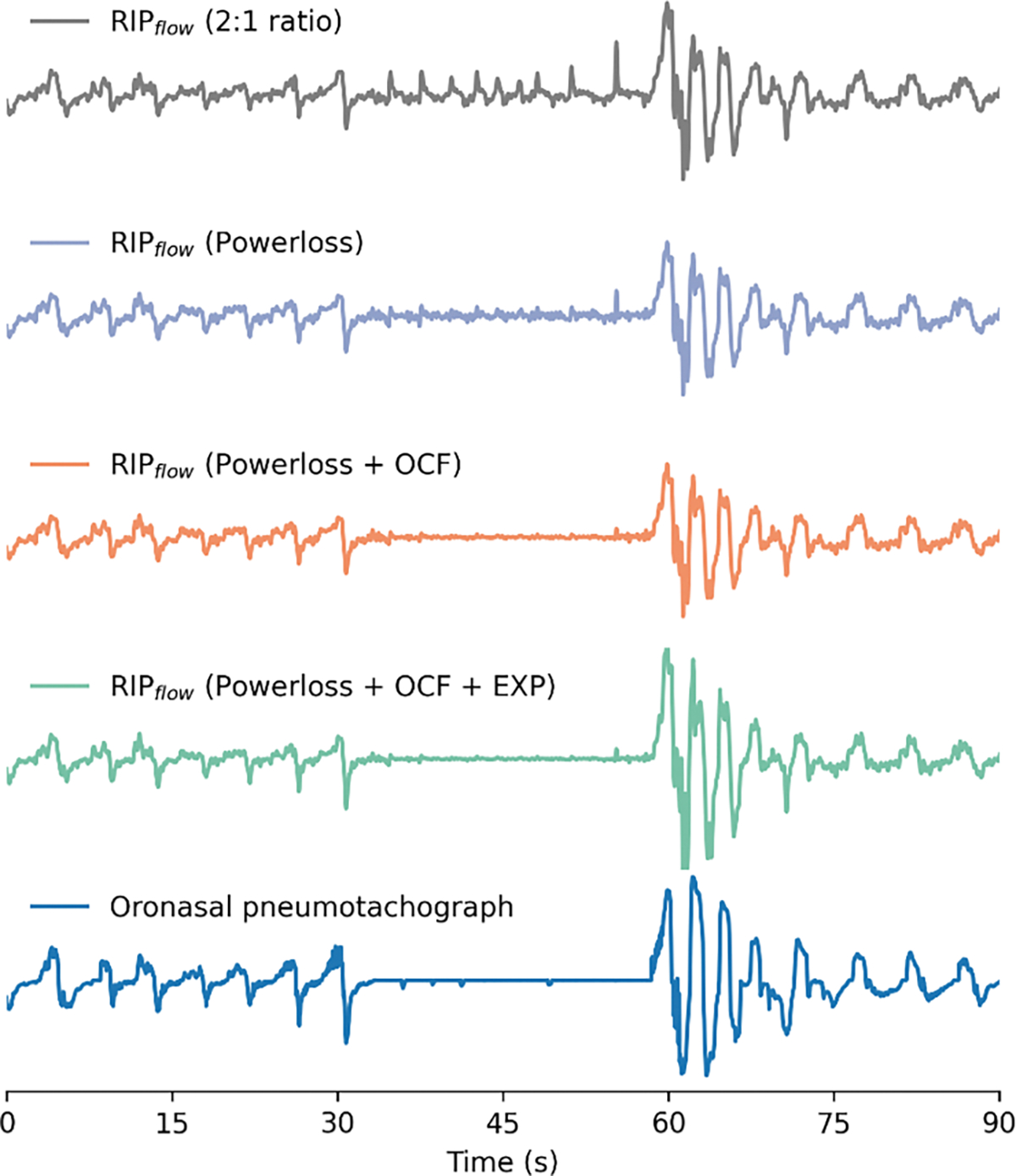
The figure illustrates a single obstructive apnea event and how the incremental application of each of the three RIP correction steps—Powerloss calibration, paradox attenuation (OCF) and exponential scaling (EXP)—moves the RIP_flow_ signal closer to the oronasal pneumotachograph gold-standard.

To provide a benchmark to evaluate the effect of different calibration and linearization techniques, performance of this three step RIP correction method was compared to a reference 2:1 scaling of the abdomen and thorax [20] (without OCF or linearity correction [steps 2–3]). Fig. 3 shows how the incremental application of each of the correction steps brings the RIP_flow_ signal closer to the oronasal pneumotach gold standard.

### G. Calculating Minute Ventilation

**Fig. 4.**
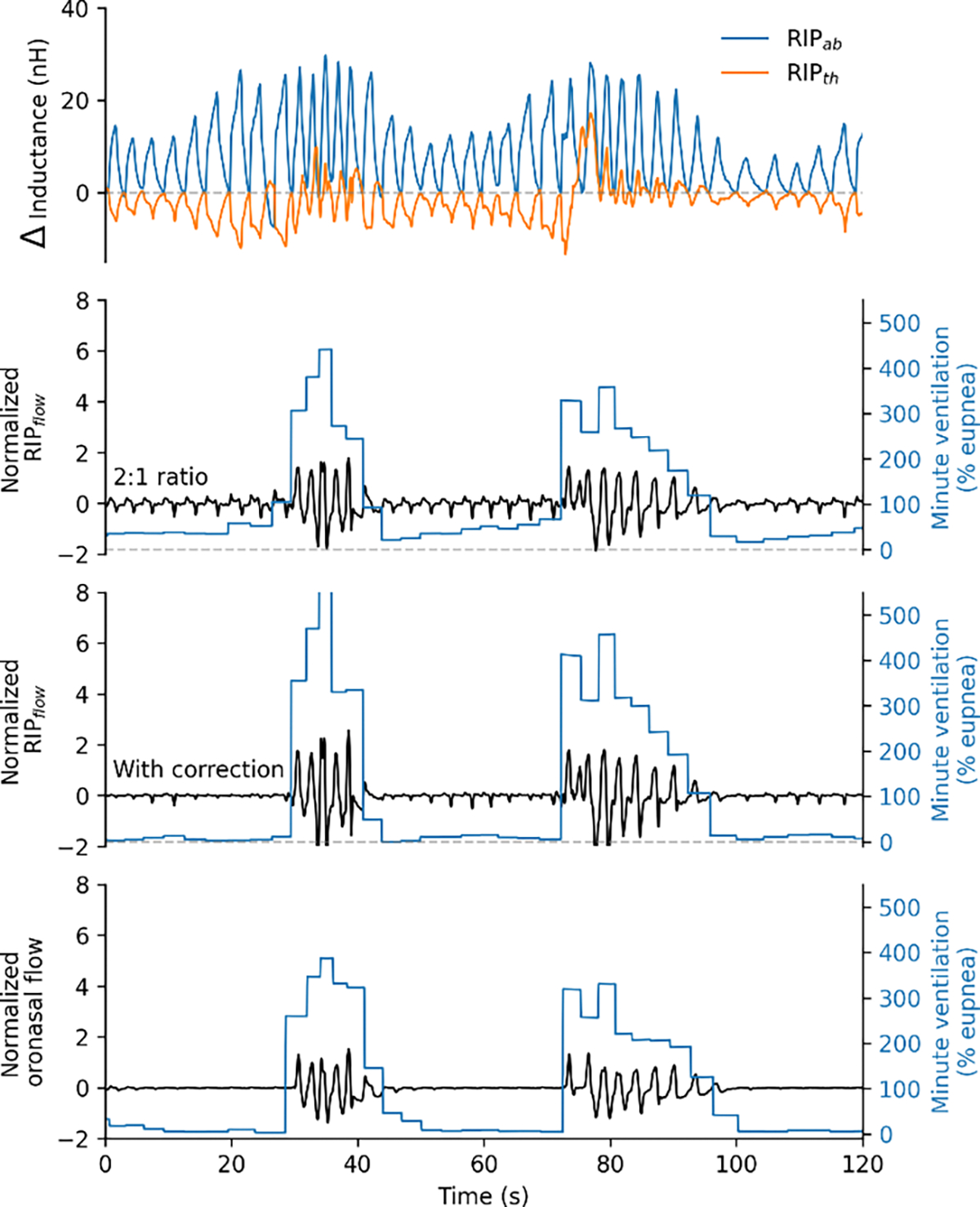
Example signals describing RIP ventilation and oronasal ventilation. Top panel, uncalibrated thorax and abdomen RIP signals that are processed and used to derive RIP flow. The signal baseline has been subtracted to improve visualization. The three lower panels depict different flow signals (black) and their corresponding minute ventilation trace (blue). First, a benchmark RIP flow signal using 2:1 ratio calibration is depicted. Second, RIP flow signal with correction applied. Third, the reference oronasal pneumotachograph flow signal. The Figure illustrates how the correction approach suppresses the residual flow during apnea, bringing the corrected RIP-based minute ventilation signal closer to the oronasal gold standard.

For the detection of respiratory events using PSG, transient reductions in flow are considered as a percentage reduction from a local baseline level, e.g. hypopneas requiring a ≥30% reduction in flow [5]. Ventilation is calculated as a product of tidal volume (arbitrary units) and respiratory rate and normalized using a local baseline. The local baseline is the mean ventilation in a 7-minute moving window [21]. A ventilation value of 100% describes mean levels (eupnea [22]), and a value of 70% describes a hypopneic breath [21], [22], [23]. The resulting signal thereby tracks proportional changes in minute ventilation. An example is shown in Fig. 4. This approach was used to calculate both RIP ventilation and oronasal ventilation for all available breaths detected during sleep.

### H. Validating RIP Ventilation

The method evaluated RIP-based minute ventilation on three levels: breath level, event level, and patient (trait) level. Briefly: 1) For individual breath analysis, breaths from all patients were pooled, and RIP ventilation values were compared against oronasal pneumotach values (gold standard). 2) For individual event analysis, RIP ventilation was used to calculate the “depth” of each respiratory event (see below). Breaths from all patients were pooled, and event depth values were compared against the value taken from the gold standard (see below). 3) Patient-specific values or “traits”, namely average event depth and ventilatory burden (see below) were calculated using RIP ventilation (single value per patient) and compared against the gold standard.

For breath-level analysis, concordance was assessed specifically for small breaths (≤70%_eupnea_, analogous to hypopnea criteria), to address the concern that RIP may overestimate ventilation during obstruction. Large breaths (≥130%_eupnea_) and medium breaths (70–130%_eupnea_) were also assessed separately. We emphasize that for application in assessment of OSA traits, the depth of the troughs and the height of the peaks in the minute ventilation signal are of particular interest.

For event-level analysis, we sought to examine respiratory events regardless of any specific desaturation or arousal criteria. Hence, for this approach, hypopneas and apneas were autoscored using the oronasal pneumotach signal (all events where ventilation dropped <30% below the local mean level for >10 seconds, see verification of autoscoring approach in the Supplementary Material). *Event depth* was calculated for each event as 100% minus the “nadir ventilation”; e.g., an event depth of 30% represents a 30% reduction in ventilation and an event depth of 100% signifies a total cessation of breathing.

For patient-level analysis, the minute ventilation signal was used to derive two advanced OSA severity measures that are not typically available in routine sleep studies due to their need for quantitative signal processing: 1) *average event depth* is a surrogate measure of upper airway collapsibility and a phenotypic biomarker of more severe OSA [24]; and 2) *ventilatory burden* as a measure that combines the event depth, duration, and frequency of respiratory events and is the ventilatory analogue to hypoxic burden [25]. Ventilatory burden has been found to predict OSA-related adverse cardiovascular outcomes with risks comparable to that of the hypoxic burden [11]. *Average event depth* was calculated as published previously (mean depth calculated from the ensemble-synchronized ventilation signal) [10]. *Ventilatory burden* was taken as the product of this average event depth, mean event duration, and the event frequency (i.e. AHI). In addition, patient-level measures of: loop gain, arousal threshold, collapsibility, and muscle compensation were explored [19], [21], [22], [26].

### I. Statistical Analysis

To assess breath-level concordance, primary analysis quantified median bias for small breaths, both without and with “RIP correction” applied. Analysis was repeated for large breaths. In principle, breath level analysis focused on minimization of bias, with the major goal of providing optimal patient-level measures. Absolute error was defined as the absolute difference between RIP and oronasal ventilation; relative error was also reported as a percentage of oronasal ventilation (omitting data with oronasal ventilation <5%_eupnea_ to avoid unstable estimates). Non-parametric summary measures of error were chosen (median bias and median absolute error) to interpret data in the presence of outliers. Plots of RIP versus the average of RIP and oronasal ventilation, along with Bland-Altman plots of relative differences, were created to illustrate the bias and dispersion between these two methods across a range of breath amplitudes. Plotting involved sorting the data into 30 bins on the x-axis with a resolution of 10%_eupnea_. For event-level concordance, primary analysis quantified mean bias and intraclass correlation (ICC(2,1) [27]) between RIP-based event depth and the gold standard, both with and without RIP correction applied. The improvement in ICC with RIP correction was assessed by paired differences of ICC. To mitigate any effects of non-normally distributed residuals, 95% confidence intervals were derived using bootstrapping with 10,000 iterations. A significant improvement was indicated if the lower bound of the 95% confidence interval (CI) for the paired differences of ICC was above zero. Plots of RIP-versus-oronasal event depth were used to illustrate median and interquartile range of RIP-derived values across multiple levels of the oronasal gold standard (20 bins).

For patient-specific measure concordance, the primary analysis quantified mean bias and ICC between RIP-based measures and the gold standard, with and without RIP correction. The improvement in ICC with RIP correction was evaluated as described above. Plots of RIP-versus-oronasal measures (i.e. estimated versus actual) and Bland-Altman absolute difference (oronasal ventilation on x-axis [28], [29]) were also provided to describe agreement. Bland-Altman plots using the average of the two measures on the x-axis were included in the Supplementary Material.

Statistical analyses were performed in Python 3.11, with NumPy 1.23.5 [15].

## III. Results

### A. Participants

Characteristics of the 89 patients are detailed in Table 1. Most participants were white (n = 68 [76.4%]) and male (n = 68 [76.4%]), with an average (SD) age of 53.6 (11.7) years and a mean (SD) body mass index (BMI) of 33.1 (6.6) kg/m^2^. The average AHI was 34.9 (24.32) events/hour and average sleep time was 6.2 (1.1) hours.

### B. Constant calibration periods

The number of constant calibration periods detected by the algorithm ranged from 5 to 96 per patient, with a mean of 37. A new k-value was calculated for each period and substantial inter- and intra-individual variability was observed. The mean k-value from the Powerloss method was 0.62 (SD: 0.10) and a mean within-patient standard deviation was 0.09 (SD: 0.05). Histograms illustrating these distributions, along with a comparison to the QDC method, are shown in Figure S3 in the Supplementary Material.

### C. Breath analysis

**Fig. 5.**
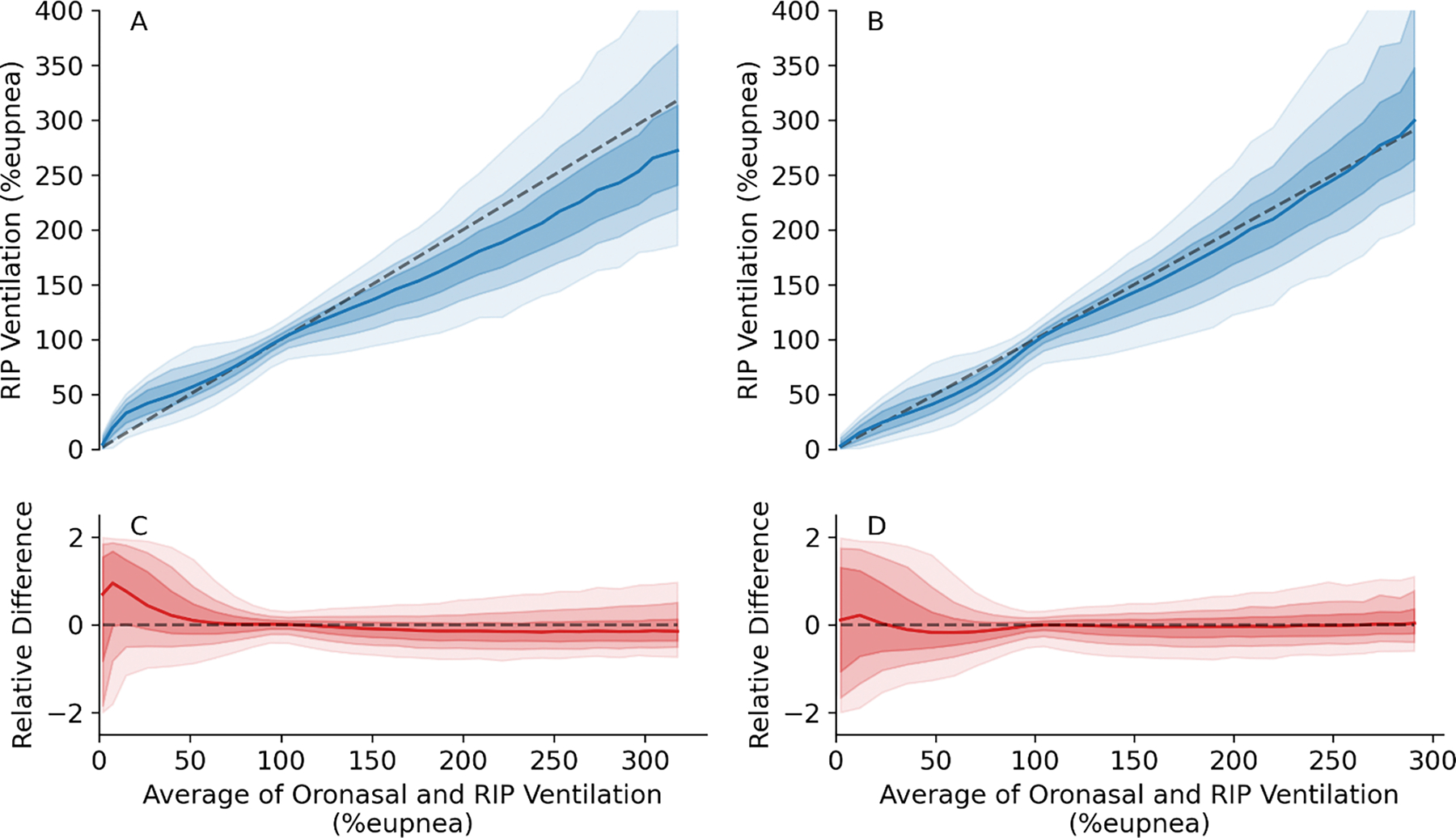
Effects of the RIP correction on RIP measurement of oronasal ventilation. The data were binned on the x-axis (N=30, step size=10%) and median and interquartile range of RIP ventilation for N=495,631 breaths plotted against the average of RIP and oronasal ventilation. Figures A and C show the results from the benchmark method using 2:1 ratio for calibration. Figures B and D show the results from the RIP correction method. In figures A and B, the dashed lines are the line of unity y=x, the blue lines show the median values, and the shaded regions show the interquartile ranges (darker) and 75th and 90th percentile (lighter). In figures C and D, the red line shows the median error (RIP minus oronasal ventilation) against the average of RIP and oronasal ventilation. The figures show how the benchmark method systematically underestimates ventilation for large breaths (>130%_eupnea_) as well as the overestimated small breaths (<70%_eupnea_), RIP correction improves this.

A total of 495,631 breaths across 89 patients were available to compare RIP ventilation against the oronasal measurement. Breath-level agreement across a range of ventilation from 0 to 300%_eupnea_ is shown in Fig. 5. Notably, compared to the 2:1 benchmark, RIP correction resulted in median RIP ventilation visibly closer to the line of unity (Fig. 5A and B) without affecting the dispersion. Without RIP correction, small breaths were overestimated, and large breaths were underestimated; RIP correction attenuated these biases (see also relative errors in Fig. 5C and D).

Overall, RIP correction markedly attenuated the overestimation bias in small breaths (N=150,214) from the median of 12.21%_eupnea_ (28% relative bias) for the benchmark method to 1.51%_eupnea_ (3%). For large breaths (N=97,728) the underestimation bias was attenuated from 32.23%_eupnea_ (17%) to 21.23%_eupnea_ (12%). However, RIP correction resulted in greater underestimation bias for medium sized breaths, from 0.41%_eupnea_ (0%) to 4.60%_eupnea_ (5%). Results of the incremental application of the three components of *RIP correction* are detailed in Table 2. Notably, *median absolute error* for all breath sizes remained similar, i.e. systematic bias was reduced while dispersion was unchanged.

### D. Event analysis

**Fig. 6.**
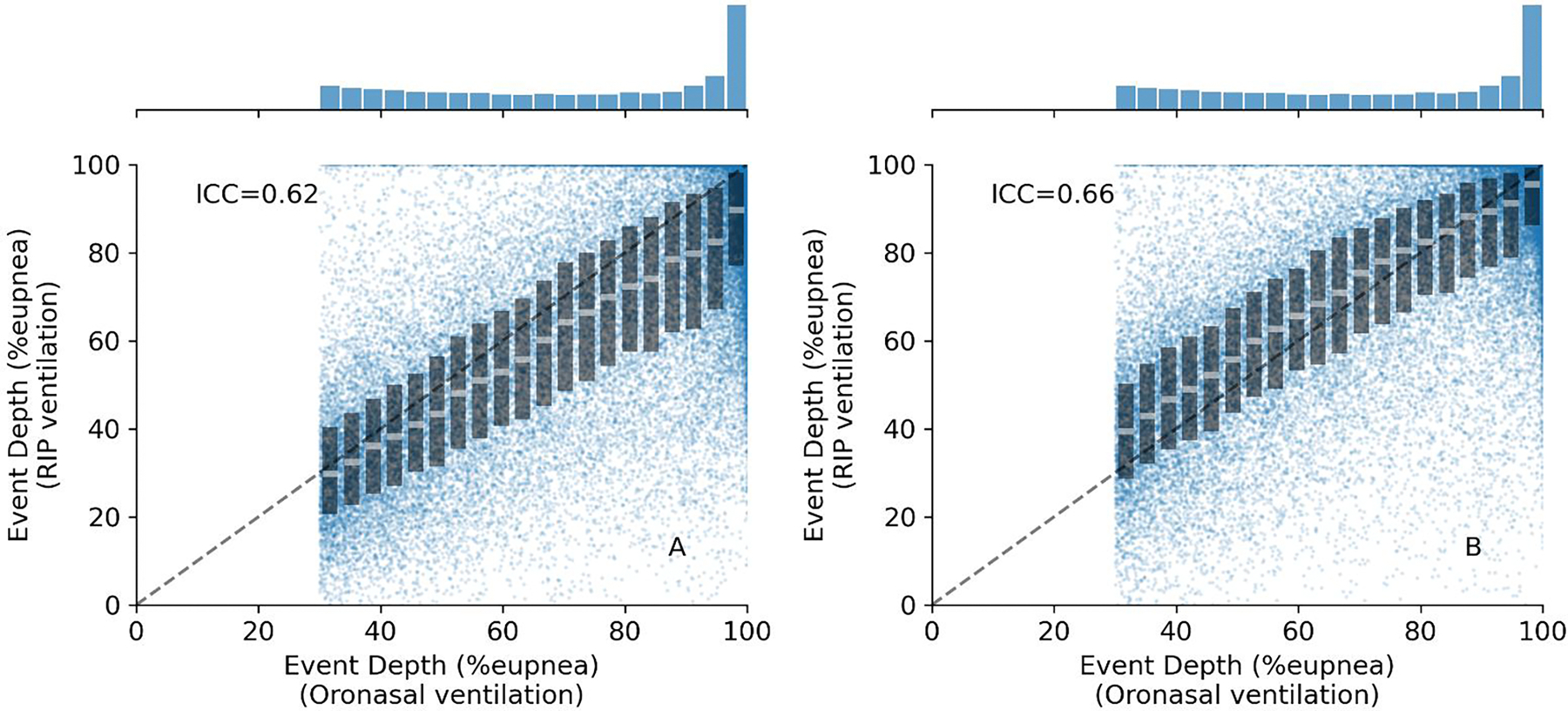
Comparison of RIP vs. oronasal ventilation measures of event depth. Event depth is defined by the magnitude of the reduction in ventilation during scored respiratory events (typically >30% by clinical definition). Individual event measures of “event depth” are shown for all N= 34,497 events without and with RIP correction (A and B respectively). The median and Interquartile range of 20 evenly spaced ventilation bins (step size=3.5%) is superimposed over individual-event data. The dashed line describes the line of identity.

RIP-derived event depth exhibited meaningful bias (underestimation) without RIP correction: 9.06 (SD: 20.73) %_eupnea_ (N = 34,497 respiratory events). With RIP correction, this underestimation bias was reduced to 1.00 (SD: 20.12) %_eupnea_. RIP-derived event depth values were concordant with those from oronasal ventilation based on ICC [95% CI] = 0.66 [0.64, 0.67] with RIP correction. Without RIP correction, ICC [95% CI] was 0.62 [0.62, 0.63] (Fig. 6); this increase in ICC was significant (95%CI of ΔICC: [0.02, 0.05]).

### E. Trait analysis

**Fig. 7.**
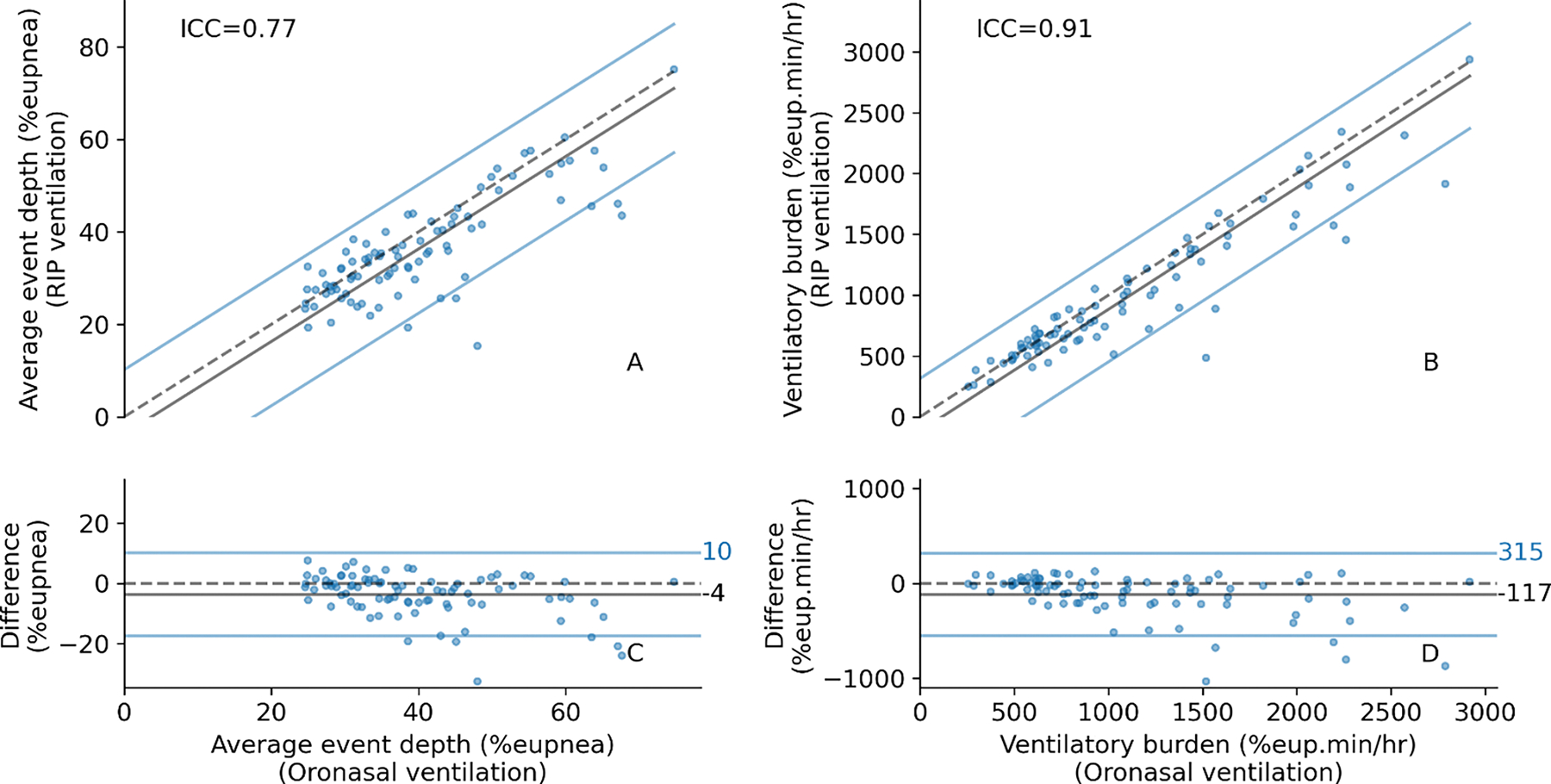
Comparison of RIP vs. oronasal ventilation measures of average event depth and ventilatory burden. Each datapoint represents a full sleep study of a single patient (N=89 patients). The dashed lines on the scatter plots (A and B) are the line of identity (y=x). The Bland-Altman bias is defined as RIP minus oronasal ventilation, and the limits of agreement are bias ± 1.96SD (C and D). The bias and limits of agreement are projected onto the scatterplots. Intraclass correlation (ICC) values are shown on the scatter plot. The plots are organized as follows: Average event depth scatterplot and corresponding Bland-Altman plot (A and C) and similarly, Ventilatory burden scatterplot and corresponding Bland-Altman plot (B and D).

Patient-specific OSA severity measures (traits)—namely *average event depth* and *ventilatory burden*—compared favorably against the gold standard: The ICC [95% CI] for average event depth and ventilatory burden were 0.77 [0.63, 0.86] and 0.91 [0.85, 0.95], respectively. These values were increased with RIP correction, up from 0.57 [0.45, 0.67] (95%CI of ΔICC: [0.15, 0.26]) and from 0.80 [0.72, 0.86] (95%CI of ΔICC: [0.08, 0.14]) respectively. Moreover, with RIP correction, systematic underestimation bias for average event depth was attenuated from 9.49 (SD: 6.80) %_eupnea_ to 3.66 (SD: 6.97) %_eupnea_. Likewise, for ventilatory burden, the underestimation bias was attenuated from 275 (SD: 241) %_eupnea_.min/hr to 116 (SD: 218) %_eupnea_.min/hr (see Fig. 7 and Figure S4 in the Supplementary Material). RIP correction did not significantly alter ICC for the estimates of loop gain, collapsibility, or compensation, with ICC values of 0.75, 0.88, and 0.64 respectively after correction (before: 0.73, 0.89, 0.68). However, ICC was significantly improved for arousal threshold estimates, increasing from 0.74 to 0.84 (95%CI of ΔICC: [0.05, 0.15]), see table S1 and Figures S5-S7 in the Supplementary Material.

## IV. Discussion

The current study developed and validated a technique for quantifying ventilation using RIP for use in overnight polysomnographic sleep studies in patients with OSA. A three-step processing approach was developed to mitigate various challenges encountered in calculating ventilation from breathing movements. These techniques reduce breath-level and event-level bias while error dispersion was largely unaffected. Accompanying the reduced bias, we observed meaningfully improved concordance for patient level measures of event severity, namely event depth and ventilatory burden. We demonstrated that it is feasible to use RIP to continuously assess ventilation during sleep and to use it to generate advanced ventilation-based measures of OSA severity. In the context of concerns about reliability of nasal pressure based airflow estimation, our study shows that RIP can be used to provide independent estimates that will be of particular interest in situations where nasal airflow signals are absent or compromised [7], [9]. Previous RIP analysis approaches primarily employed simple “sum”-based (linear calibration), using a ratio of thorax and abdomen signals that is designed to minimize the RIP-derived flow signal amplitude in the presence of paradoxical breathing movements (i.e. ISOCAL [14] and QDC [13]). However, our study reveals that simply adding thoracic and abdominal signals together, even with an optimal ratio, is not sufficient to prevent overestimation of ventilation during small breaths. Our study demonstrated that two additional approaches were useful. First, further suppression of flow amplitude for breaths with negatively correlated thoracic and abdominal movement signals, based on the degree of correlation (The OCF step of our calibration approach), improved the concordance between RIP and gold standard ventilation, see Table 2. Second, the application of a nonlinear power-law scaling also markedly improved the concordance of the approach. These findings highlight that simple sum-like linear calibration techniques, commonly used in clinical recording systems for scoring events, can underestimate the severity of airflow reduction, and can be corrected with more advanced processing.

There is increasing interest in quantitative estimation of ventilation during sleep, not only for the automated detection of respiratory events, but also for the goal of providing insight into both OSA severity [10], [11], [30] and its underlying etiology [10], [19], [21], [22], [26]. Our technique, despite its modest error on a breath-by-breath basis, reduced bias in the estimation of the amplitude of reduction in ventilation in individual respiratory events (9 to 1 %eupnea). Consequent to the improved bias, the method provided for greater agreement in patient level measures including *average event depth* [10] and *ventilatory burden* [11] (ICC=0.77 “good” and ICC=0.91 “excellent” respectively [27], up from 0.57 “moderate” and 0.80 “good” respectively without RIP correction). Our analysis also supported favorable estimation of ventilation-based estimates of OSA etiology, with RIP-corrected ICCs for arousal threshold, loop gain, collapsibility between 0.75 and 0.88 (“good”) and an ICC for compensation of 0.64 (“moderate”). In a recent study where high loop gain was investigated as a predictor of the blood pressure response to CPAP therapy [31], the authors validated a RIP-derived loop gain against nasal pressure and found an ICC of 0.58. Additional use of these parameters to predict responses to therapies or prevent adverse outcomes warrants further exploration.

We considered the following strengths and limitations. First, the EXPO parent study targeted recruitment across a broad range of obesity levels to ensure a sufficient sample of individuals with BMI ≥ 35 and those ≤ 25 kg/m^2^, such that our results are likely generalizable across a broad range of BMI levels. We acknowledge, however, that the parent study sought patients with AHI ≥ 15 events/hour; thus, despite our inclusion of those with AHI > 5 events/hour in our analysis, we may be underrepresenting individuals with mild OSA (AHI between 5 and 15). Moreover, our study population was predominantly male (76%) and white (76%); further validation in women and/or non-White groups may be helpful. Exploratory analysis revealed that sex had minimal impact on breath-level results (e.g., small breath bias: 1.8 % eupnea in men vs. −0.9 % in women). Similarly, performance did not meaningfully differ between obese (BMI ≥ 30) and non-obese participants (e.g., small breath bias: 1.9 vs. 0.9 in non-obese participants; n = 29). The modest sample size in our study (n=89, due to the technical complexity and invasiveness of the oronasal pneumotachograph measurement) did not allow for a statistically robust evaluation of algorithm performance across OSA severity levels. Second, as with all measurement devices, RIP ventilation accuracy is expected to be affected by sensor placement; when the two RIP belts are improperly placed such that both belts are measuring the same compartment, reduced accuracy is expected. In contexts where belt placement cannot be monitored by a technician, automated methods for ascertaining belt placement would be beneficial. Fourth, steps 2 and 3 of the RIP correction method required the tuning of a handful of specific parameters, which were adjusted using a subset of the validation data. This approach risks overfitting the method to this dataset, potentially providing optimistic results and reducing its applicability to other datasets. Validating the method on a different dataset would be useful. Additionally, the algorithm for detecting movements was tuned pragmatically rather than systematically optimized. The thresholds were visually validated to ensure reliable performance, but not formally benchmarked. The resulting distribution of detected movements (mean 37, range 5–96 per patient per night) appeared physiologically reasonable, though direct comparisons with existing studies were not possible due to the absence of a standardized definition of “movements”. Fifth, the results are not necessarily expected to apply to heavily filtered RIP or non-RIP based respiratory movement measures. Sixth, the current study did not directly compare nasal pressure and RIP-derived measures; further studies comparing the separate or combined utility for predicting responses to therapies or adverse outcomes of OSA are warranted.

## V. Conclusion

This study demonstrates that with appropriate calibration and correction, RIP signals can provide accurate estimates of ventilation during sleep and enable the derivation of advanced OSA severity metrics, including event depth and ventilatory burden. The proposed method reduced systematic bias at both the breath and event levels and improved concordance with pneumotach-derived measures. These findings show that RIP, already a standard component of PSG, can be enhanced to yield reliable ventilation-based metrics, particularly when nasal airflow measurements are absent or unreliable.

## Supplementary Material

RIP Supplement

## Figures and Tables

**Table 1.** PATIENT CHARACTERISTICS. CONTINUOUS VARIABLES ARE DESCRIBED BY MEANS AND (STANDARD DEVIATION). CLINICAL PARAMETERS ARE BASED ON MANUAL SCORING.

Characteristic	Value

N	89
Age, yr.	53.6 (11.7)
Gender, M:F	68:21
Body mass index, kg/m2	33.1 (6.6)
Race/Ethnicity	
White, N	68
Black, N	14
Asian, N	5
Other, N	2
Total sleep time (TST), h	6.18 (1.1)
REM, %TST	10.86 (7.68)
N1, %TST	25.61 (18.705)
N2, %TST	51.9 (15.96)
N3, %TST	11.59 (9.29)
Supine position, %TST	49.71 (33.91)
Apnea-hypopnea index, events/hr.	34.89 (24.32)
Central Apnea Index, events/hr.	0.70 (1.68)
Fraction of hypopneas	0.65 (0.32)

**Table 2.** Ventilation prediction error across different breath sizes for the different calibration and linearization techniques. Small breaths are defined as breaths where oronasal ventilation < 70%_eupnea_ and large breaths defined as breaths where oronasal ventilation > 130%_eupnea_ and medium breaths are defined in between. Bias is defined as RIP minus oronasal ventilation, where positive values indicate overestimation of ventilation. Number of small breaths; N=150,214, number of medium breaths; N=247.689, number of large breaths; N=97,728. Total number of breaths N=495,631.

		Median Bias %_eupnea_				Dispersion Median Absolute Error %_eupnea_			
	
	Method	Small breaths	Medium breaths	Large breaths	All breaths	Small breaths	Medium breaths	Large breaths	All breaths

Benchmark	2:1	12.21 (28%)	−0.42 (0%)	−32.23 (17%)	0.56 (1%)	16.38 (46%)	10.40 (11%)	42.66 (22%)	15.04 (17%)

RIP correction steps	(A) Powerloss	11.00 (25%)	−0.44 (0%)	−29.24 (16%)	0.53 (1%)	15.19 (43%)	9.71 (10%)	40.32 (21%)	13.97 (16%)
	(B) Powerloss + OCF	9.25 (22%)	−0.39 (0%)	−27.88 (15%)	0.25 (1%)	14.44 (43%)	9.73 (10%)	39.80 (21%)	13.73 (16%)
	(C) Powerloss + OCF + EXP	1.51 (3%)	−4.60 (5%)	−21.23 (12%)	−3.35 (6%)	12.95 (42%)	12.62 (13%)	43.05 (23%)	15.54 (19%)
