# Supplementary material for: Respiratory Inductance Plethysmography to Quantify Changes in Ventilation in Obstructive Sleep Apnea
Source: IEEE Trans Biomed Eng. Author manuscript; Available in PMC 2026 May 1. (PMC12989205; doi:10.1109/TBME.2025.3618403)
Supplement: RIP Supplement [file NIHMS2147753-supplement-RIP_Supplement.docx]

# Supplementary materials

These are supplementary materials for a manuscript titled 'Respiratory Inductance Plethysmography to Quantify Changes in Ventilation in Obstructive Sleep Apnea', by E. Finnsson et al. Published in IEEE Transactions on Biomedical Engineering, 2025.

## Study participants

Adult male and female participants were recruited from the Hospital of the University of Pennsylvania, USA and Landspítali University Hospital, Iceland. Before participating, each participant signed a written informed consent. The study carefully selected participants based on the following inclusion and exclusion criteria.

Inclusion Criteria: Participants must be proficient in English, have access to a telephone, be between the ages of 18 and 80, and possess an Apnea-Hypopnea Index (AHI) of either ≥15 or ≤5. They should also have a Body Mass Index (BMI) of ≥35 or ≤25, maintain a stable medical history over the past two months, abstain from alcohol for 24 hours prior to a sleep study, and cease using CPAP or oral appliances for 48 hours before the sleep study.

Exclusion Criteria: Candidates are disqualified if they are unable or unwilling to consent to participation, face restrictive time constraints, or are diagnosed with sleep disorders other than Obstructive Sleep Apnea (OSA), such as central sleep apnea, insomnia, obesity-hypoventilation syndrome, restless leg syndrome, PLM disorder, or narcolepsy. Additional disqualifying factors include having more than 20 periodic limb movements per hour with arousals, prior surgery for OSA, clinical instability such as recent severe medical conditions or surgeries (e.g., myocardial infarction, congestive heart failure, unstable angina, arrhythmias, severe COPD, or recent cancer diagnosis), conditions or treatments affecting the structure or function of the upper airway or gastrointestinal tract (e.g., muscle weakness, trauma, surgery, radiotherapy, chemotherapy), regular use of sedatives or related medications, the presence of metal parts that are incompatible with MRI procedures, or being pregnant.

## Autoscoring evaluation and AHI

Autoscoring of respiratory events using the oronasal pneumotach signal demonstrated good agreement with manual scoring, achieving an ICC of 0.85 for the AHI. To ensure consistency in AHI comparisons, autoscored hypopnea events were excluded if they were not associated with manually identified desaturations or arousals. On average, the autoscoring algorithm overestimated the AHI by approximately 10 events per hour relative to manual scoring.

To mitigate the bias induced by autoscoring, we compared the AHI derived from a 2:1 ratio calibration and the corrected RIP signal with the oronasal mask autoscored AHI. RIP correction reduced the bias in AHI from –3.7 to –0.5 events/hour, while the limits of agreement expanded slightly from ±15.1 to ±15.7 events/hour. Agreement between the autoscored mask AHI and both RIP-derived AHIs remained high, with ICC unchanged at 0.94.

## RIP correction

The manuscript outlines a three-step process aimed at calibrating and linearizing Respiratory Inductance Plethysmography (RIP) signals to enhance the accuracy of RIP flow estimates. The process includes: first, a linear calibration using the power loss method; second, a non-linear suppression technique to eliminate periods of paradoxical breathing; and third, the application of a power-law scaling factor to improve linearity when compared to the gold standard oronasal mask flow. Each of these steps will be described in detail in the following paragraphs. This structured approach is designed to refine the measurement of respiratory flow using RIP technology.

### The powerloss method

The RIP belts measure changes in the circumference of the thorax and abdomen. Assuming a constant height of these two compartments, the RIP signals can be thought of as measurements of volume. Therefore, a ventilatory flow waveform signal can be calculated from the RIP signals “RIP_flow_” by using the time derivative of the calibrated RIP signal, Eq. S1, where $dRIP_{Ab}$ and $dRIP_{Th}$ are the time derivatives of the abdominal and thoracic RIP signals, respectively and $k$ is the calibration constant.

| $RIP_{flow}= (1-k) dRIP_{Th}+ k dRIP_{Ab}$ | (S1) |
| --- | --- |

The original approach to RIP calibration (i.e. finding the constant *k*) was based on a method proposed by Konno and Mead^1^, who demonstrated that *k* can be found by performing an iso- volume breathing maneuver, inducing paradoxical movement of the abdomen and thorax, and choosing the value of *k* such that the measured amplitudes of the abdominal and thoracic excursions cancel out. This is called iso-volume maneuver calibration or ISOCAL^1^, and was originally validated on waking patients.

In a PSG sleep study, breathing movements are typically recorded using RIP belts. However, calibrating these belts using the iso-volume maneuver is problematic during sleep studies because the calibration constant can change as the sleeping patient moves and the belts shift positions. Consequently, it is not standard practice to perform an iso-volume bio-calibration prior to a sleep study. A major goal of this research is to quantitatively assess ventilation using clinical data where the iso-volume maneuver was not performed. Therefore, we must derive the calibration constant from spontaneous breathing signals observed throughout the night. In this study, we discuss three methods for recalibrating RIP belts from spontaneous breathing. For each method, the calibration constant is recalculated following any automatically detected movements, ensuring accuracy despite any sensor movement.

First, as a reference method, Banzett et. al. showed that simply adding the two RIP belts together in a fixed ratio of 2:1 (chest-to-abdomen) could provide a reasonable default calibration^2^.

Second, as our preferred method, we employ the powerloss method by calculating the optimal *k* as that which minimizes the variability in the calibrated RIP flow signal. More precisely, *k* is found to minimizes the ratio of the root mean square (RMS) of the calibrated RIP_flow_ signal to the sum of the RMS of the separate thoracic and abdominal flow signals, Eq. S2. In principle, when the calibration constant is tuned appropriately, the paradox component seen in each separate signal ([1−*k*]RIP_th_, [*k*]RIP_ab_) is maximally subtracted out in the calibrated sum.

| $\min_{k} \frac{RMS((1-k)\times dRIP_{th}+k\times dRIP_{ab})}{RMS((1-k)\times dRIP_{th}) + RMS(k\times dRIP_{ab})} where 0\leq k\leq1$ | (S2) |
| --- | --- |

Third, as an alternative method for calibration, we examined the qualitative diagnostic calibration (QDC) technique^3^. In the QDC method, *k* is chosen to scale the thoracic and abdominal volume waveforms such that they have similar variability. In our implementation we calculated separate abdominal and thoracic “tidal volumes” (peak to trough, labeled V_th_ and V_ab_) and identified the value of *k* that provided equal standard deviations i.e. SD([k-1]V_th_) = SD([k]V_ab_). The method is not our preferred approach because there are valid concerns that QDC may only work provided constant or quasi-constant tidal volume^4^, and the accuracy of QDC has been challenged^5^.

Additionally, alternative use of the actual oronasal ventilation signal to provide a “true” calibration coefficient did not provide a substantially reduced level of error. This suggests that the linear calibration methods provide results that are close to the upper limit of RIP calibration performance and therefore additional (e.g. non-linear) methods are required to improve performance further.

### Paradox attenuation

To address the problem of paradoxical breathing movements not being completely cancelled in RIP signals, we developed a technique called the RIP Paradox Overestimation Correction Factor (OCF). This approach specifically targets the subset of breaths most impacted by paradoxical movements, characterized by a low Pearson correlation coefficient (r), between the thoracic and abdomen RIP signals, ranging from -1.0 to -0.8.

The construction of the OCF involved developing a sigmoidal function designed to suppress the RIP flow during pronounced paradoxical movements. This function is constrained to values between a specified offset and 1, with three adjustable parameters: the midpoint x_0_=0.034, the steepness k=31.0 and the offset y_min_=0.22 the relationship between which is detailed in Eq. S3. The application of the OCF on the RIP flow is described in Eq. S4, where ‘corr’ is a function that calculates the breath-wise Pearson's correlation coefficient between the signals from the two belts, see Fig. S2.

| $ocf\left( r \right)=\left( 1 - y_{min} \right)\left( \frac{1}{1+e^{-k\left( (r+1)/2-x_{0} \right)}} \right)^{2}+y_{min}$ | (S3) |
| --- | --- |

| $RIP_{flow}^{'}=RIP_{flow}\times ocf(corr(RIP_{ab}, RIP_{th})$ | (S4) |
| --- | --- |

Fig. S1A displays the ratio of oronasal to RIP ventilation as a function of Pearson's r, illustrating that this ratio remains close to 1 for r values from -0.6 to 1 but decreases for values lower than -0.6, indicating that the RIP tends to overestimate ventilation when the bands move out of phase. The OCF is visually represented in this figure by a blue line superimposed on the boxplots.

We analyzed data from a specific subset of small breaths (N=20,707), identified as having paradoxical movements with Pearson's r between -1.0 and -0.8. The application of the OCF significantly reduced the median bias in these cases from 7.8%eupnea to 0.9%eupnea. Fig. S1B illustrates the bias in RIP ventilation estimates before and after applying the OCF, shown in gray and blue boxes, respectively. The figure highlights how the OCF effectively reduces the bias for r values at or below -0.8, without impacting estimates where Pearson's r is higher.

| 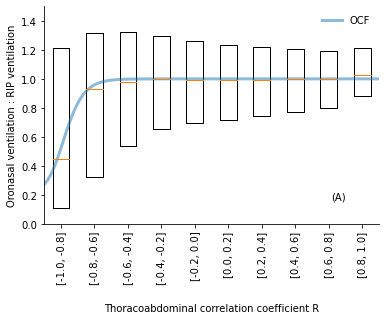 | 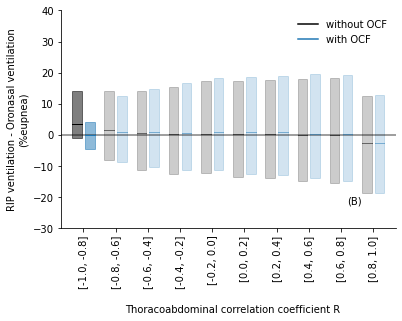 |
| --- | --- |

Fig. S1 During breaths with paradox (obstructive periods), RIP tends to overestimate ventilation when compared to oronasal ventilation. (A) The ratio of the oronasal ventilation to RIP ventilation. The figures show that the RIP overestimates ventilation during out-of-phase periods. The observed error follows a sigmoidal shape; a sigmoid was fit to the data and referred to as overestimation correction factor (OCF). The OCF suppresses RIP ventilation during out-of-phase periods. (B) Application of the OCF reduced the absolute bias observed. We emphasize that the bias was present regardless of calibration strategy (2:1 ratio, powerloss, QDC); we used powerloss calibration but not power-law scaling for this analysis.


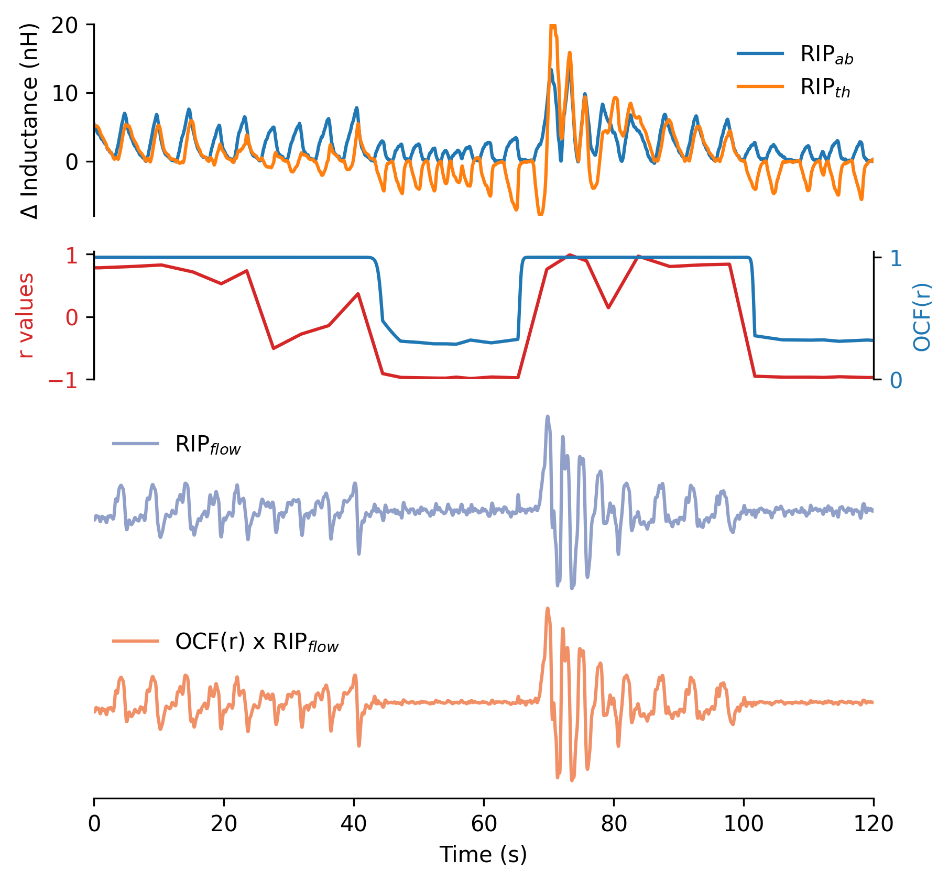


Fig. S2 The top panel displays the RIP (Respiratory Inductance Plethysmography) signals, with the baseline removed to clearly show paradoxical movements. In the middle panel, the derived metrics are showcased: the Pearson correlation coefficient in red and the overestimation correction factor (OCF) in blue. This panel highlights that the OCF is activated only under conditions of severe paradox. The bottom panel illustrates RIP_flow_ calculated by two different methods: depicted in blue is the RIP_flow_ derived using the powerloss method without the OCF, and in orange, the RIP_flow_ with the OCF incorporated, effectively minimizing residual flow during episodes of obstructive apnea.

### Exponential scaling

To address the empirically observed non-linear relationship between RIR-derived and oronasal-derived ventilation, namely, underestimation of ventilation for large breaths and overestimation of ventilation during small breaths we applied a power-law scaling factor with an exponent of 1.17 see Eq. S5.

| $RIP_{flow}^{'}=sign\left( RIP_{flow} \right)\left\vert RIP_{flow} \right\vert^{1.17}$ | (S5) |
| --- | --- |

In the study, we chose to scale the RIP_flow_ signal directly rather than a ventilation signal. This is because we wanted to isolate the RIP_flow_ signal generation from its specific use of getting a ventilation signal. Exponential scaling of a flow signal necessitates a zero-drift condition in the signal, indicating no flow when the value is zero.

The necessity for an exponential scaling factor is not immediately clear. It is possible that the simple two-compartment model, representing the abdomen and thorax, fails to capture all aspects of volume displacement, and that a third direction of expansion becomes significant only during very deep breaths.

### Distribution of k-values

A single calibration constant (k) is often assumed in sleep studies. However, our data reveal significant inter- and intra-individual variability in k-values (defined in Eq. S1), suggesting that a static calibration may not capture dynamic respiratory changes during sleep. Fig. S3 shows the means and standard deviations of k-values for the study cohort, highlighting this variability and underscoring the need for an adaptive calibration approach. Before applying the different calibration approaches the signals are initialized to a 2:1 (chest-to-abdomen) ratio.


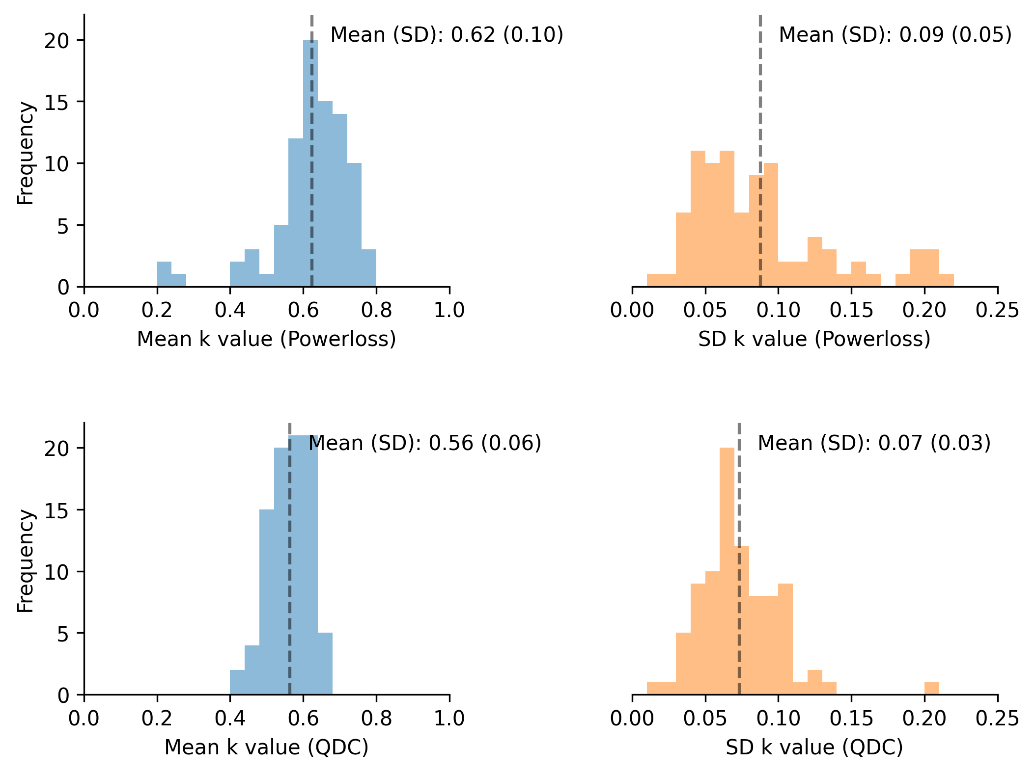


Fig. S3 Distribution of mean and standard deviation (SD) k-values across the study cohort for the Powerloss and QDC calibration methods—initialized using a 2:1 (chest-to-abdomen) ratio. Each histogram represents the distribution of k-value statistics computed per patient, with the left column showing the mean k-values and the right column showing their SDs. The dashed lines indicate the overall mean of each distribution. These results highlight substantial inter- and intra-individual variability in k-values, suggesting that a single static calibration constant may not fully capture dynamic respiratory changes during sleep.

## Trait estimation

The study aimed to find methods for estimating patient level traits from RIP signals. To this end, a three-step calibration and correction approach was developed and applied. Agreement with gold standard measures of flow was assessed using Bland-Altman analysis and Intraclass Correlation Coefficient (ICC). The following chapter provides more details on the statistical results reported in the manuscript.

### Bland-Altman analysis

The manuscript features a comparison of RIP-derived patient-level traits against the oronasal gold standard. In the manuscript, we present the gold standard on the x-axis; however, recognizing the ongoing debate over this approach to Bland-Altman plotting, we include in Fig. S4 and Fig. S5 conventional Bland-Altman plots for each trait, using the mean of the reference and estimate on the x-axis.

| 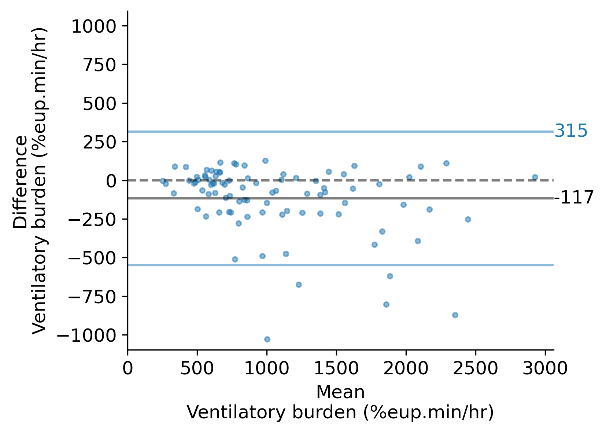 | 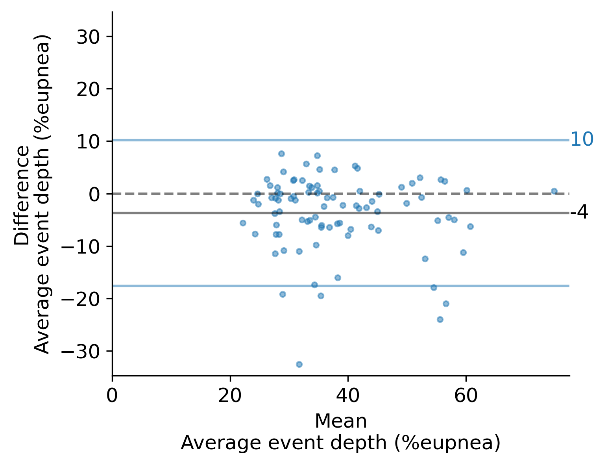 |
| --- | --- |

Fig. S4 Bland-Altman plot. Left: Ventilatory Burden; Right: Average Event Depth. Differences are calculated as RIP minus Oronasal. Bias is annotated in black, and the 95% limits of agreement are marked in blue.

| 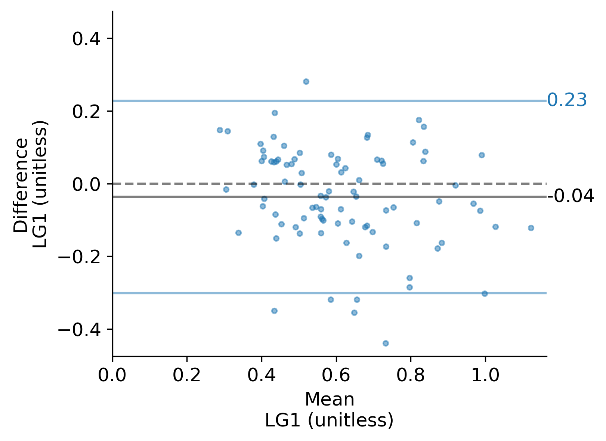 | 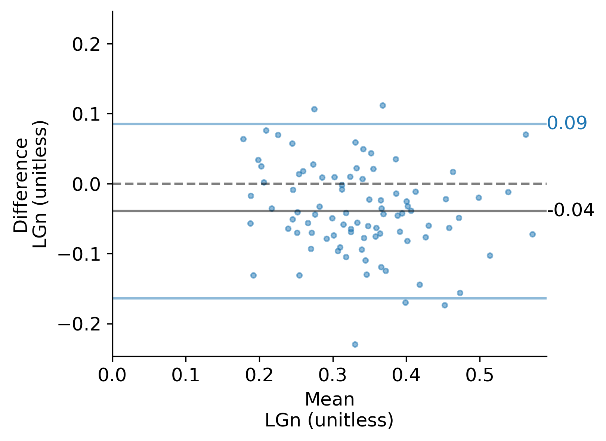 |
| --- | --- |
| 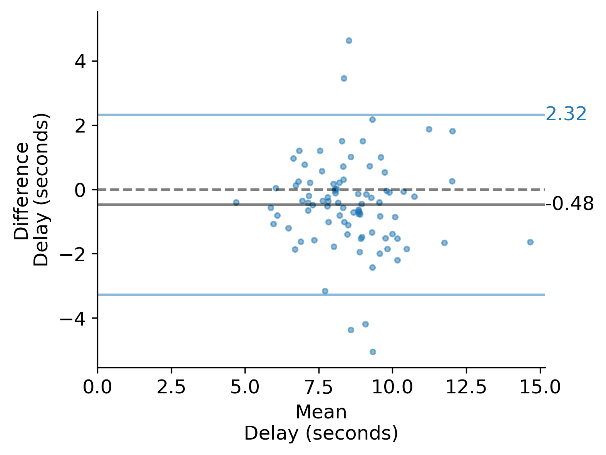 | 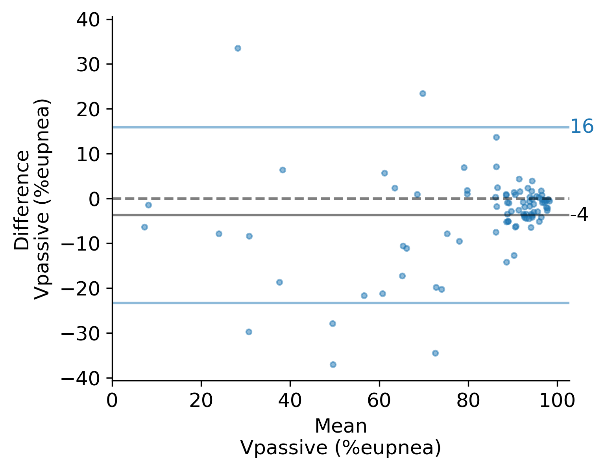 |
| 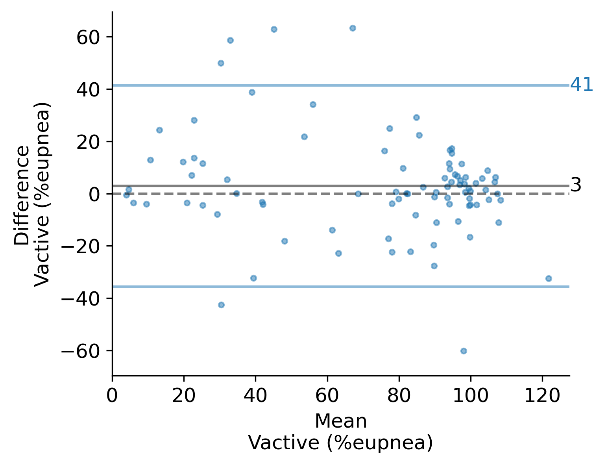 | 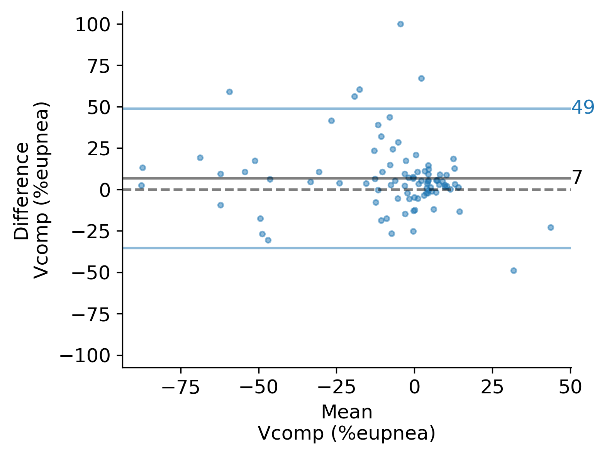 |
| 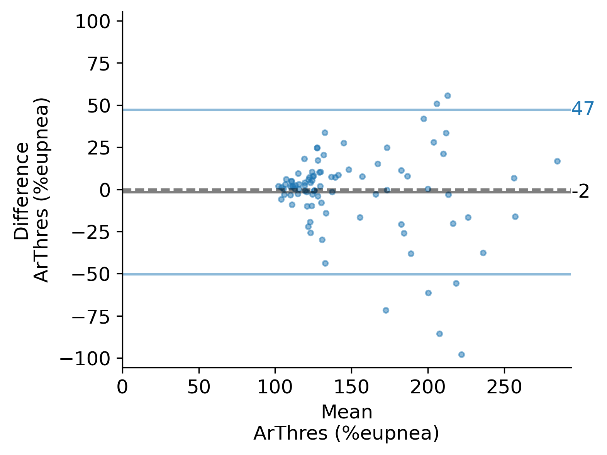 | 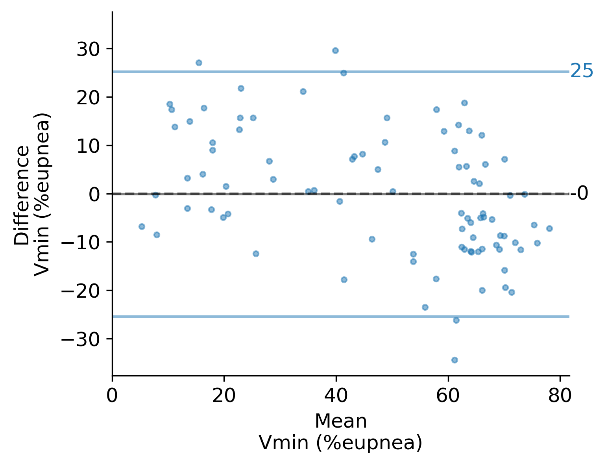 |

Fig. S5 Physiological Traits Analysis. Displayed from top left to bottom right: Loop gain at 1 cycle per minute (LG1), loop gain at the natural frequency (LGn), circulatory delay (delay), ventilation at eupneic drive (Vpassive), ventilation at arousal threshold (Vactive), upper airway compensation (Vcomp = Vactive - Vpassive), arousal threshold (ArThres), and ventilation at nadir drive (Vmin). Differences are calculated as RIP minus Oronasal. Bias is marked in black, and the 95% limits of agreement are highlighted in blue. (ArThres), ventilation at nadir drive (Vmin). Differences are calculated as RIP minus Oronasal. Bias is annotated in black, and the 95% limits of agreement are marked in blue.

### Endotypic traits

The main variables of interest for the context of the manuscript were average event depth, a surrogate measure for upper airway collapsibility, and ventilatory burden, a novel measure of OSA severity. In addition, analysis was performed to estimate endotypic traits from the ventilation data. Traits were algorithmically approximated from PSG data using PUPpy^6^, a cloud based version of PUP^7–9^. The concordance between the two measures is shown in Fig. S6 and Fig. S7.

TABLE S1 Patient level trait agreement with and without RIP correction. The star (*) marks a significant improvement in ICC post correction.

|  | ICC Without correction | ICC With correction | Delta ICC | Target mean (SD) | Without correction mean (SD) | With correction mean (SD) | Without correction mean error (SD) | With correction mean error (SD) |
| --- | --- | --- | --- | --- | --- | --- | --- | --- |
| Average event depth* | 0.56 | 0.77 | 0.20  [0.15, 0.26] | 39.95 (11.56) | 30.46  (9.39) | 36.25 (10.86) | -9.48 (6.83) | -3.68 (7.00) |
| Ventilatory burden* | 0.80 | 0.91 | 0.11  [0.08, 0.15] | 1110.61 (607.38) | 836.20 (457.33) | 994.55 (540.77) | -274.28 (241.26) | -115.93 (217.40) |
| LG1 | 0.73 | 0.75 | 0.02  [-0.04, 0.07] | 0.63  (0.21) | 0.59  (0.17) | 0.60  (0.18) | -0.04 (0.13) | -0.04 (0.13) |
| LGn | 0.66 | 0.70 | 0.04  [-0.03, 0.10] | 0.36  (0.10) | 0.31  (0.08) | 0.32  (0.09) | -0.04 (0.07) | -0.04 (0.06) |
| Delay | 0.64 | 0.61 | -0.02  [-0.12, 0.06] | 8.80  (1.70) | 8.28  (1.76) | 8.32  (1.62) | -0.51 (1.41) | -0.48 (1.41) |
| Vpassive | 0.89 | 0.88 | -0.01  [-0.07, 0.04] | 83.22 (20.35) | 85.24 (17.54) | 79.46 (22.64) | 2.04  (8.73) | -3.76 (9.87) |
| Vactive* | 0.78 | 0.83 | 0.05  [0.01, 0.08] | 71.29 (35.16) | 82.14 (26.66) | 74.24 (31.34) | 10.78 (18.38) | 2.94 (19.43) |
| Vcomp | 0.68 | 0.64 | -0.04  [-0.09, 0.02] | -11.86 (27.26) | -3.07 (22.38) | -5.15 (24.47) | 8.72 (18.57) | 6.65 (21.17) |
| ArThres* | 0.74 | 0.84 | 0.10  [0.05, 0.15] | 149.53 (46.05) | 133.17 (31.69) | 147.98 (42.01) | -16.36 (24.65) | -1.56 (24.58) |
| Vmin* | 0.79 | 0.84 | 0.05  [0.00, 0.09] | 48.19 (25.05) | 55.86 (19.70) | 48.10 (19.60) | 7.62 (12.90) | -0.11 (12.80) |

| 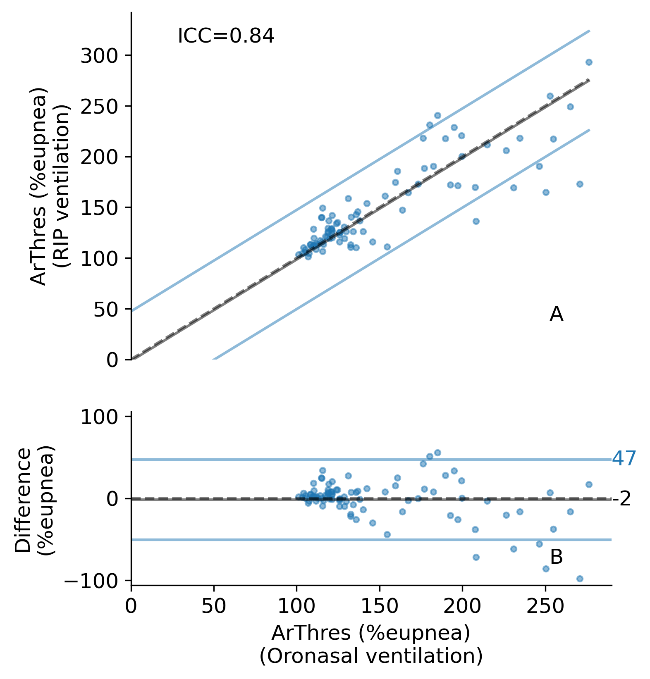 | 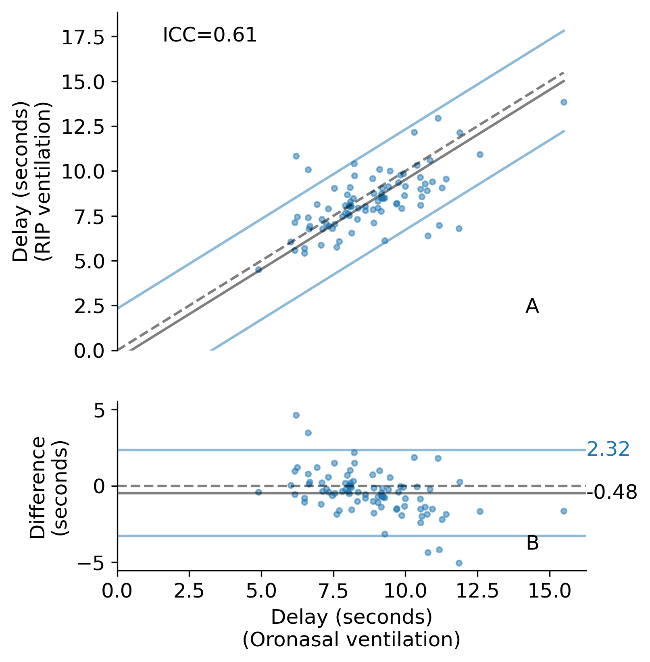 |
| --- | --- |
| 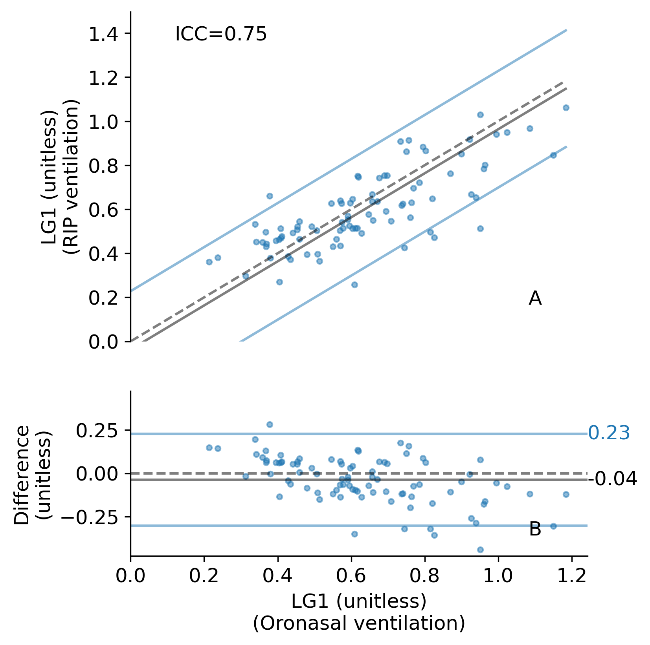 | 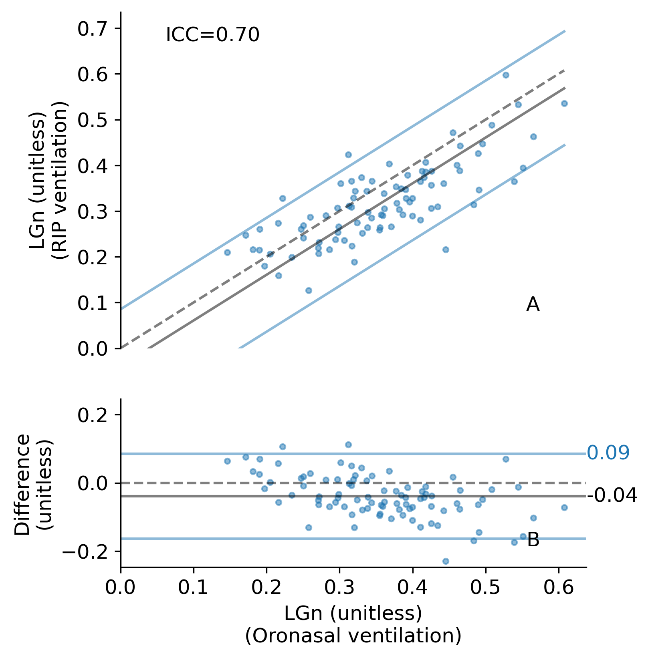 |

Fig. S6 Physiological Traits Analysis—arousability and ventilatory control. Displayed from top left to bottom right: Arousal threshold (ArThres), circulatory delay (delay), Loop gain at 1 cycle per minute (LG1), and loop gain at the natural frequency (LGn). Each figure features two sections, A and B, which share an x-axis. Section A is a scatterplot displaying traits derived from RIP on the y-axis against their oronasal counterparts on the x-axis. Section B shows a plot of the differences (calculated as RIP minus Oronasal) against the oronasal target. In both plots, bias is annotated in black, and the 95% limits of agreement are highlighted in blue.

| 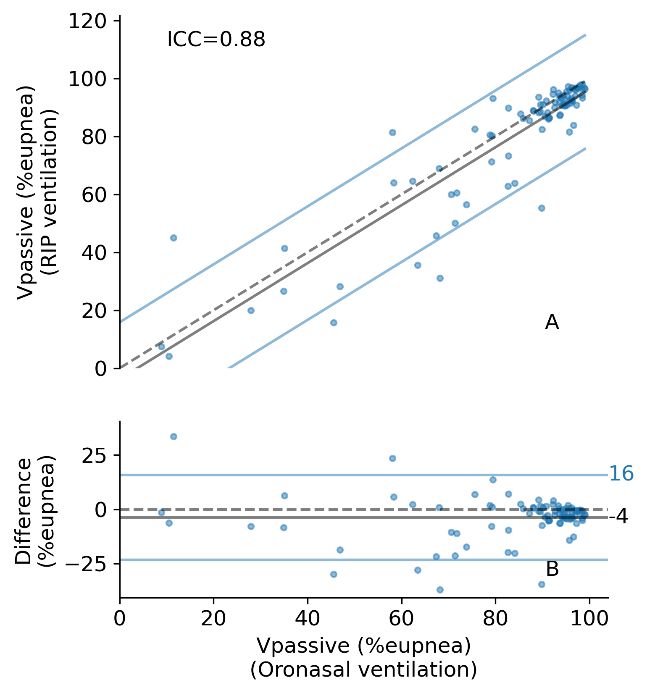 | 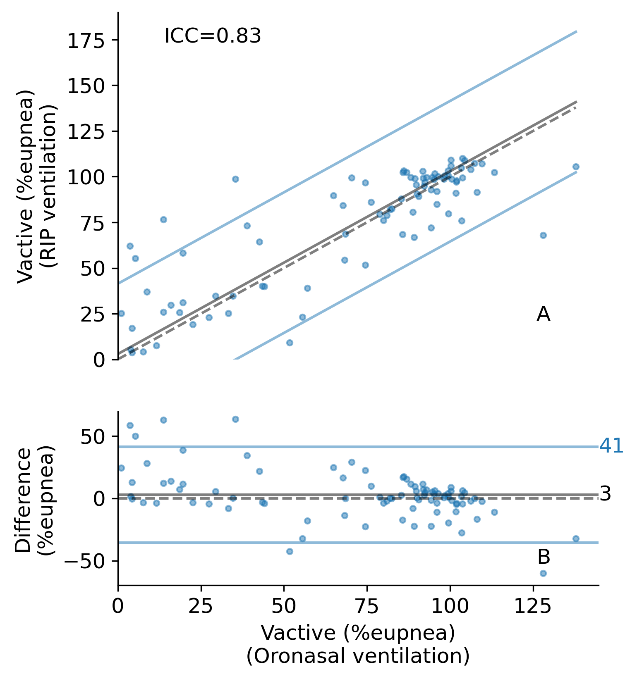 |
| --- | --- |
| 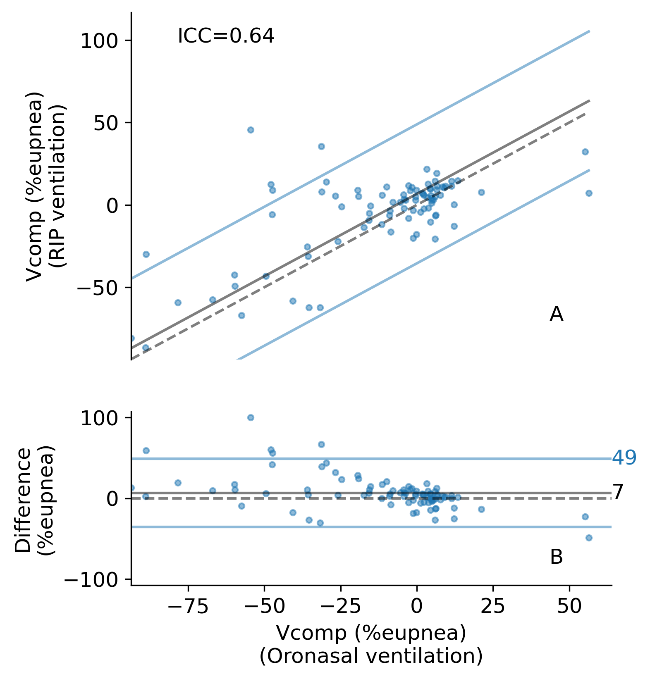 | 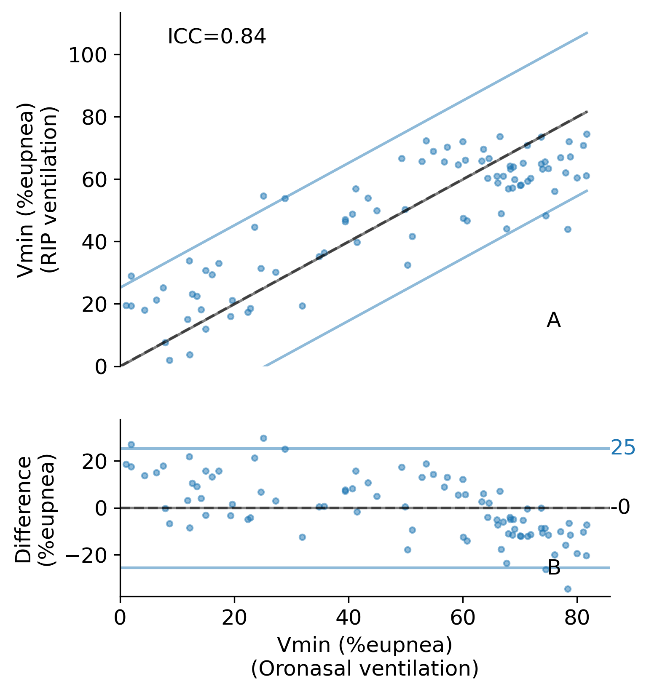 |

Fig. S7 Physiological Traits Analysis—upper airway physiology. Displayed from top left to bottom right: ventilation at eupneic drive (Vpassive), ventilation at arousal threshold (Vactive), upper airway compensation (Vcomp = Vactive - Vpassive) and ventilation at nadir drive (Vmin). This figure features two sections, A and B, which share an x-axis. Section A is a scatterplot displaying traits derived from RIP on the y-axis against their oronasal counterparts on the x-axis. Section B shows a plot of the differences (calculated as RIP minus Oronasal) against the oronasal target. In both plots, bias is annotated in black, and the 95% limits of agreement are highlighted in blue.

# References

1. Konno K, Mead J. Measurement of the separate volume changes of rib cage and abdomen during breathing. *J Appl Physiol*. 1967;22(3):407-422. doi:10.1152/jappl.1967.22.3.407

2. Banzett RB, Mahan ST, Garner DM, Brughera A, Loring SH. A simple and reliable method to calibrate respiratory magnetometers and Respitrace. *J Appl Physiol*. 1995;79(6):2169-2176. doi:10.1152/jappl.1995.79.6.2169

3. Sackner MA, Watson H, Belsito AS, et al. Calibration of respiratory inductive plethysmograph during natural breathing. *J Appl Physiol 1985*. 1989;66(1):410-420.

4. De Groote A, Paiva M, Verbandt Y. Mathematical assessment of qualitative diagnostic calibration for respiratory inductive plethysmography. *J Appl Physiol*. 2001;90(3):1025-1030. doi:10.1152/jappl.2001.90.3.1025

5. Barbosa RCC, Carvalho CRF de, Moriya HT. Pletismografia respiratória por indutância: estudo comparativo entre calibração por manobra de isovolume e calibração qualitativa diagnóstica em voluntários saudáveis avaliados em diferentes posturas. *J Bras Pneumol*. 2012;38(2):194-201. doi:10.1590/S1806-37132012000200008

6. Finnsson E, Ólafsdóttir GH, Loftsdóttir DL, et al. A scalable method of determining physiological endotypes of sleep apnea from a polysomnographic sleep study. *Sleep*. 2021;44(1):zsaa168. doi:10.1093/sleep/zsaa168

7. Terrill PI, Edwards BA, Nemati S, et al. Quantifying the ventilatory control contribution to sleep apnoea using polysomnography. *Eur Respir J*. 2015;45(2):408-418. doi:10.1183/09031936.00062914

8. Sands SA, Terrill PI, Edwards BA, et al. Quantifying the Arousal Threshold Using Polysomnography in Obstructive Sleep Apnea. *Sleep*. 2018;41(1). doi:10.1093/sleep/zsx183

9. Sands SA, Edwards BA, Terrill PI, et al. Phenotyping Pharyngeal Pathophysiology using Polysomnography in Patients with Obstructive Sleep Apnea. *Am J Respir Crit Care Med*. 2018;197(9):1187-1197. doi:10.1164/rccm.201707-1435OC
